# Coumarin-Annulated Ferrocenyl 1,3-Oxazine Derivatives Possessing In Vitro Antimalarial and Antitrypanosomal Potency

**DOI:** 10.3390/molecules26051333

**Published:** 2021-03-02

**Authors:** Mziyanda Mbaba, Laura M. K. Dingle, Ayanda I. Zulu, Dustin Laming, Tarryn Swart, Jo-Anne de la Mare, Heinrich C. Hoppe, Adrienne L. Edkins, Setshaba D. Khanye

**Affiliations:** 1 Department of Chemistry, Faculty of Science, Rhodes University, Makhanda 6140, South Africa; mziyanda.mbaba@uct.ac.za (M.M.); azulu50@gmail.com (A.I.Z.); 2 Department of Biochemistry and Microbiology, Faculty of Science, Rhodes University, Makhanda 6140, South Africa; lauradingle25@hotmail.co.uk (L.M.K.D.); dustinlaming89@gmail.com (D.L.); g10s2905@campus.ru.ac.za (T.S.); j.delamare@ru.ac.za (J.-A.d.l.M.); h.hoppe@ru.ac.za (H.C.H.); a.edkins@ru.ac.za (A.L.E.); 3 Biomedical Biotechnology Research Unit, Rhodes University, Makhanda 6140, South Africa; 4 Centre for Chemico- and Biomedicinal Research, Rhodes University, Makhanda 6140, South Africa; 5 Division of Pharmaceutical Chemistry, Faculty of Pharmacy, Rhodes University, Makhanda 6140, South Africa

**Keywords:** organometallic, bioorganometallic, ferrocene, coumarin, oxazine, malaria, trypanosomiasis, cancer, *Plasmodium falciparum*, *Trypanosoma brucei*, mode of action

## Abstract

A tailored series of coumarin-based ferrocenyl 1,3-oxazine hybrid compounds was synthesized and investigated for potential antiparasitic activity, drawing inspiration from the established biological efficacy of the constituent chemical motifs. The structural identity of the synthesized compounds was confirmed by common spectroscopic techniques: NMR, HRMS and IR. Biological evaluation studies reveal that the compounds exhibit higher in vitro antiparasitic potency against the chemosensitive malarial strain (3D7 *P. falciparum*) over the investigated trypanosomiasis causal agent (*T. b. brucei* 427) with mostly single digit micromolar IC_50_ values. When read in tandem with the biological performance of previously reported structurally similar non-coumarin, phenyl derivatives (i.e., ferrocenyl 1,3-benzoxazines and α-aminocresols), structure-activity relationship analyses suggest that the presence of the coumarin nucleus is tolerated for biological activity though this may lead to reduced efficacy. Preliminary mechanistic studies with the most promising compound (**11b**) support hemozoin inhibition and DNA interaction as likely mechanistic modalities by which this class of compounds may act to produce plasmocidal and antitrypanosomal effects.

## 1. Introduction

Malaria and trypanosomiasis are protozoal infections that are endemic in tropical regions. Both diseases are vector-borne and are transmitted through the bite of infected female *Anopheles* mosquitos and tsetse flies, respectively. Of the five known protozoal species of the genus *Plasmodium* (i.e., *P. falciparum*, *P. vivax*, *P. knowlesi*, *P. ovale* and *P. malariae*), *P. falciparum* and *P. vivax* are responsible for the most cases of malaria, with the former accounting for more than 99% of infections in the African continent in 2018 alone, according to the World Health Organisation [1,2]. Infection of the human host with protozoal *Trypanosoma* species causes human African trypanosomiasis (also known as sleeping sickness) [3]. Although these diseases are preventable and curable, courtesy of cutting-edge medical technologies of the modern day and the concerted efforts by both the public and private sector, they still pose a great burden to the healthcare sector in developing economies and threaten many lives. Particularly, initiatives such as the Medicines for Malaria Venture (MMV) and Drugs for Neglected Diseases Initiative (DNDi) are among the leading programmes of the past decades that spear-headed drug discovery innovation directed towards the cause of eradicating tropical diseases, including malaria and trypanosomiasis [4,5]. Despite these efforts, the causal agents of these diseases are increasingly becoming resistant to current clinical drugs employed for their treatment. For instance, emergence of *P. falciparum* strains that are resistant to the highly efficacious artemisinin-combination therapies (ACTs), introduced by WHO as a failsafe for malaria resistance [6], have already been recorded [7]. Hence, research towards the design and biological assessment of novel therapeutic agents that can elude or impede the development of clinical resistance by pathogens is an active field in contemporary drug discovery.

**Figure 1:**
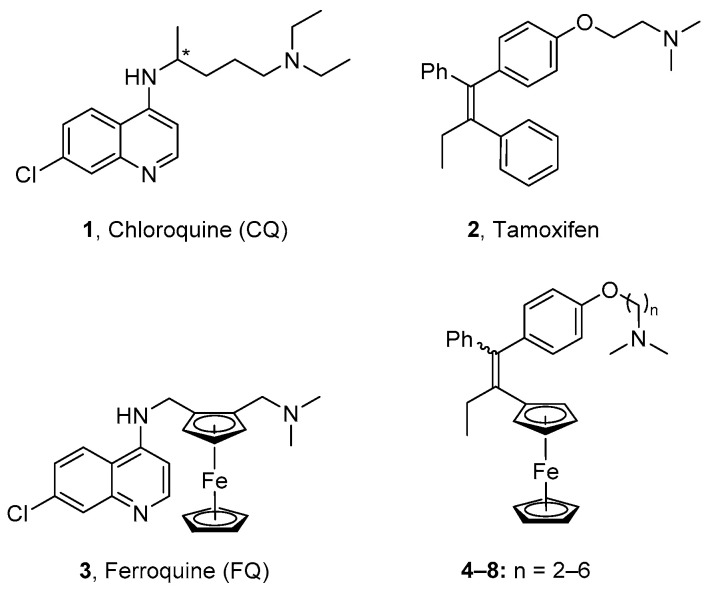
Chemical structures of chloroquine (**1**), tamoxifen (**2**) and their ferrocenyl derivatives ferroquine (**3**) and ferrocifens (**4**–**8**).

Medicinal and bioorganometallic chemists are turning to the use of nonconventional organometallic complexes, employing contemporary drug discovery strategies that incorporate mechanistically distinct pharmacophores, as potential alternative medicinal agents with unique modes of action that can possibly overcome the scourge of clinical resistance [8,9,10]. Ferrocene is the prime example in the application of organometallic complexes in medicinal chemistry due to its attractive physicochemical attributes such as high lipophilicity, reversible redox character and ability to generate membrane disruptive reactive oxygen species (ROS) in biological media [11,12]. The incorporation of ferrocene into the known antimalarial drug scaffold, chloroquine (**1**, CQ), and the framework of an anticancer agent, tamoxifen (**2**), respectively, yielded an ingenious antimalarial clinical candidate, ferroquine (**3**, FQ), and a cohort of promising pre-clinical anticancer leads, ferrocifens (**4**–**8**) (Figure 1) [13,14,15,16]. These organometallic compounds display superior biological performance to their organic parent drugs on the respective diseases and possess distinct, complementary mechanisms of action, which are attributed to the presence of ferrocene. Consequently, the success of ferroquine and ferrocifens have inspired researchers to introduce ferrocene to other bioactive organic motifs, like quinoline [17,18,19,20], indole [21,22,23], benzimidazole [24,25] and oxanes [26,27,28], to modulate their therapeutic efficacy for a myriad of biological applications.

Coumarins are prominent heterocyclic compounds in medicinal chemistry and show a wide spectrum of biological activities including anticancer [29,30], antimalarial [31,32], antitrypanosomal [33,34], antileishmanial [35,36] and antitubercular effects [37,38]. Previously, we demonstrated that introducing ferrocene into the naturally occurring coumarin-based antibiotic, novobiocin, by replacing the benzamide side chain enhanced the in vitro antiproliferative effects of the resulting ferrocenyl derivatives, i.e., ferrobiocins, against triple-negative human breast cancer cells (HCC38 and MB-DA-231) and endowed the new compounds with antiplasmodial activity against chemosensitive 3D7 *P. falciparum* trophozoites in vitro [39,40]. Furthermore, the research groups of Yan and Zhang synthesized and evaluated the in vitro anticancer activity of ferrocenyl coumarins against several cancer cell lines with activities in the low and mid-micromolar range [41,42]. Apart from these limited accounts, there are no other reports on the biological activity of ferrocenyl coumarin derivatives as far as we know. We have also recently showed that ferrocenyl 1,3-benzoxazines, commonly utilised as precursors in the synthesis of electrochemically active polymers [43,44,45], could be repurposed as potential medicinal agents against malaria and trypanosomiasis as well as breast cancer [46]. The 1,3-oxazine moiety was found to play a crucial role in the biological activity of these compounds [46,47]. Presented with the prolific biological profile of coumarins and the encouraging results obtained from conjugating coumarin and ferrocene and the significant pharmacological role of the oxazine unit in ferrocenyl 1,3-benzoxazines, we envisaged combining these structural units, i.e., coumarin, oxazine and ferrocene, to explore the antiplasmodial and antitrypanosomal potential of the resulting hybrid compounds against the chloroquine-sensitive (CQS) 3D7 *P. falciparum* strain and *T. brucei brucei* trypomastigotes. Preliminary mechanistic studies using a representative compound as a tool suggest DNA interaction and hemozoin inhibition as possible modes of action by which the investigated conjugates may elicit biological activity.

## 2. Results

### 2.1. Chemistry

**Figure 2:**
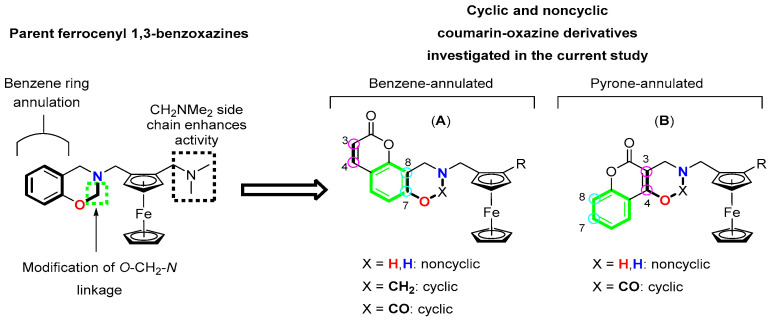
Design of ferrocenyl coumarin-oxazine hybrid compounds pursued in this study.

Given our promising findings with previously reported ferrocenyl 1,3-benzoxazines [46], as well as their non-cyclic aminocresol counterparts [47], we envisioned expanding the benzene ring in order to gain better insights into the structure-activity relationship profile of this class of compounds by replacing this unit with a privileged coumarin scaffold. Thus, the target compounds were designed to incorporate all three desired structural units, i.e., coumarin, oxazine and ferrocene, as illustrated in Figure 2. First, a plain ferrocenyl 1,3-oxazine unit bearing a *O*-CH_2_-*N* bridge was appended to the benzene ring of the coumarin nucleus (A) (Figure 2). Second, considering the postulated pharmacophoric importance of the oxazine *O*-CH_2_-*N* connectivity [48,49], we introduced a carbonyl group to produce less basic carbamate analogues, i.e., coumarin-1,3-oxazin-2-ones (B), containing a *O*-CO-*N* oxazine linkage annulated to the prone unit (Figure 2). In our literature search we noted that there are numerous examples of benzene annulated coumarin-oxazines in the literature, however, we could only find one account of the pyrone-amended counterparts [50]. In light of this, and as a secondary objective of the current study, we directed our efforts towards pursing the lesser explored pyrone-annulated congeners (B) of these compounds for biological assessment. Lastly, we varied the basic ferrocenyl methyl(dimethylamine) motif (R = CH_2_NMe_2_) known to enhance biological activity, e.g., in ferroquine [13], by removing it to produce representative compounds lacking this moiety (R = H) in order to probe its pharmacological significance.

**Scheme 1:**
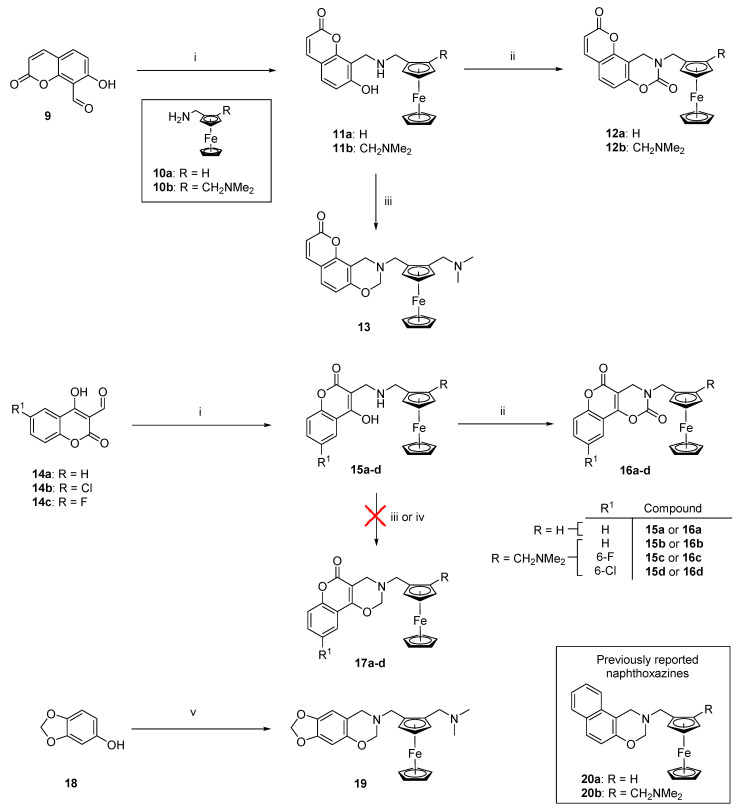
Synthesis of noncyclic and cyclic ferrocenyl coumarin-oxazine derivatives. Reaction conditions: (i) ferrocenyl amine (**10a** or **10b**, 1.0 eq.), EtOH, reflux, 5–6 h followed by NaBH_4_ (2.0 eq.), r.t. to reflux, 15–30 min; (ii) CDI (1.0 eq.), DCM, r.t., 1–2 h; (iii) paraformaldehyde (1.0 eq.), CHCl_3_, reflux, 10 h; (iv) paraformaldehyde (2.0 eq.), FeCl_3_ (10 mol%), aq. SDS (10 mol%), r.t., 12 h; (v) paraformaldehyde (2.0 eq.), CHCl_3_, reflux, 10 h.

Synthesis of the target compounds was achieved by reductive amination of formyl hydroxycoumarins **9** and **14a**–**c** with ferrocene methylamines **10a**,**b**, prepared according to literature methods [13,51,52,53,54], that was affected by sodium borohydride as the reducing agent in yields ranging between 66 and 86% (Scheme 1) [55]. Benzene-annulated hybrid compound **13** was readily obtained by cyclisation of the corresponding aminocoumarin-7-ol **11b** under reflux with paraformaldehyde [46]. However, pyrone-annulated aminocoumarin-4-ols (**15a**–**d**) did not cyclize under these conditions. The use of a previously reported SDS-mediated cyclisation procedure for pyrone-annulated coumarin-1,3-oxazines proved unsuccessful in furnishing the desired products **17a**–**d**, either [50]. The failure of the alcohols to cyclize could be a result of the non-aromaticity of the pyrone ring and effects of keto-enol tautomerism [56], both of which are not conducive for electrophilic aromatic substitution via which the reaction proceeds [57]. Cyclisation of these substrates (**15a**–**d**) was thus achieved by reaction with 1,1′-carbonyldiimidazole (CDI) to yield the corresponding less basic benzene- and pyrone-annulated coumarin-oxazin-2-ones (**12a**,**b** and **16a**–**d**, respectively) [58]. Lastly, we included a hybrid compound (**19**) containing a fused, two-ring sesamyl unit featuring a benzodioxole motif in lieu of the coumarin nucleus for comparison purposes. This was synthesized as previously described for compound **13** above employing two equivalents of paraformaldehyde instead of one [46]. For the discussion of biological results, we also included structurally related naphthoxazines **20a**,**b**, which we reported elsewhere [46], as part of the SAR elucidation campaign to gain a better understanding of the pharmacological effects of expanding the benzene ring. 

The structural identity of all the compounds was confirmed by common spectroscopic techniques: nuclear magnetic resonance (^1^H and ^13^C NMR) and high-resolution mass spectrometry (HRMS). The absence of the aldehyde signal and appearance of new ferrocenyl and methylene peaks in their distinctive regions at δ 4.51–4.01 ppm and δ 4.99–3.02 ppm, respectively, verified successful amination of formyl hydroxycoumarins (**9** and **14a**–**d**) in the ^1^H NMR spectra of products **11a**,**b** and **15a-d**. This was corroborated by ^13^C NMR data. Assignment of the proton signals was achieved by multiplet analysis of the ^1^H NMR signals as well as 2D NMR techniques: COSY, HSQC and HMBC (Supporting Information). The ^13^C NMR data of coumarin-oxazin-2-ones (**12a**,**b** and **16a**–**d**) were invaluable in the confirmation of successful cyclization of aminohydroxycoumarin substrates (**11a**,**b** and **15a**–**d**), which revealed the new CO signals around δ 147.0 ppm, characteristic of the carbamate unit. For the plain cyclic coumarin- and sesamyl-oxazines (**13** and **19**), the distinctive oxazine methylene protons resonated at δ 4.96–4.78 ppm (O-CH_2_-N) and δ 4.06–4.02 ppm (aryl-CH_2_-N) as previously observed in the literature [46].

### 2.2. Biological Evaluation

The in vitro antimalarial and trypanocidal activities of the target compounds was realised on the blood-stage CQS 3D7 *P. falciparum* trophozoites and nagana *T. b. brucei* 427 trypomastigotes, respectively. The biological evaluation results are presented in Table 1. Excluding **11b**, all the compounds were also assessed for antiproliferative effects against the HCC70 triple-negative breast cancer cell line. However, none of the tested coumarin- and sesamyl-oxazine derivatives were effective at inhibiting the growth of the evaluated cells at the tested concentration (25 µM single point screening, data not shown).

The investigated compounds were more selective for the plasmodial parasites over the trypomastigotes, with at least a 2-fold increase in activity. With the exception of **16a**, acyclic derivatives (e.g., **11a**,**b** and **15c**,**d**) exerted better potencies than their cyclic coumarin-oxazin-2-one variants (e.g., **12a**,**b** and **16c**,**d**) against both pathogens, with compound **11b** commanding the highest activities in the α-aminohydroxycoumarin series: IC_50_ = 1.7 µM (3D7) and 9.4 µM (427). This could be attributed to the effect of the intramolecular hydrogen bond between the amine (NH) and hydroxyl (OH) groups of the aminohydroxycoumarins, which has been postulated to be pivotal for biological activity in similar ferrocenyl α-aminocresols and other antiparasitic agents reported in the literature [47,59]. The plain coumarin-oxazine **13** was the most active in the whole series against the *P. falciparum* strain with an IC_50_ value of 1.0 µM, while its sesamyl congener **19** displayed activity of 4.78 µM. Naphthoxazines **20a**,**b** showed comparable antiplasmodial activities (IC_50_ = 3.39 µM (**20a**) and 4.12 µM (**20b**)) to sesamyl-oxazine **19**, and were more active than that the pyrone-annulated coumarins (**15a**–**d** and **16a**–**d**) [46]. Preliminary screening of the compounds for general toxicity effects and selectivity for parasitic cells was assessed on the mammalian cervical cancer HeLa cell line and effected at least 60% viability of these mammalian cells (see Figure S1, Supporting Information). This suggests a higher selectivity profile of the compounds for the parasitic pathogens over mammalian cells at the tested concentration.

SAR analysis shows that modification of the coumarin nucleus on the benzene ring, either by the introduction of an α-aminocresol moiety (**11a**,**b**) or annulation with an oxazin-2-one unit (**12a**,**b**), is more favourable for activity than annulation on the pyrone unit (**15a**–**d** and **16a**–**d**) (Table 1). This is further attested by the activity of the benzene-annulated sesamyl-oxazine **19** and naphthoxazines **20a**,**b**, which were many times more potent than the pyrone-modified coumarin derivatives (**15a**–**d** and **16a**–**b**). Since the pyrone unit is non-aromatic, the takeaway from this is that the phenolic oxygen atom contained in the benzene-annulated analogues is crucial for biological activity. Concerning the effect of the oxazine rings, cyclisation with the *O*-CH_2_-*N* linkage (**13**) is more desirable for activity compared to the less basic *O*-CO-*N* connection (**12a**,**b**). This emphasizes the pharmacological significance of the *O*-CH_2_-*N* linkage as previously observed for parent ferrocenyl benzoxazines [46]. Substitution at coumarin C-6 with fluorine (**15c**, IC_50_ = 8.76 µM [3D7] and 19.6 µM [427]) exerted slightly inferior activity compared to chlorine (**15d**, IC_50_ = 5.7 µM [3D7] and 5.0 µM [427]) for acyclic derivatives (**15a-d**), while this effect was reversed for the cyclic analogues (**16a**–**d**, IC_50_ = 12.55 µM (3D7) [**16c**] vs. n.a. (3D7) [**16d**]) (Table 1). On the other hand, the presence of the hydrogen atom was detrimental for activity as all compounds with this group were inactive against both parasites. Regarding the modification of the ferrocene unit, compounds containing the ferrocenyl CH_2_NMe_2_ motif on the top cyclopentadiene ring (Cp) were generally more effective than their congeners lacking this unit, signifying its pharmacological importance. In view of the superior antiplasmodial and antitrypanosomal activities of previously reported parental ferrocenyl 1,3-benzoxazines and α-aminocresols (noncyclic analogues) that were active in low and sub-micromolar range [46,47], the moderate biological performance of the ring-expanded coumarin-oxazines investigated in the current study suggests that replacing the benzene ring of the parent scaffold with a coumarin nucleus leads to reduced antiparasitic activity. Nevertheless, benzene-annulated coumarin-oxazine derivatives **11b** and **13** display similar potencies to their previously reported benzoxazine and α-aminocresol counterparts in the same order of magnitude [46,47], indicating that the coumarin nucleus is reasonably tolerated for activity when annulated on the benzene unit. The structural bulk of coumarin could also impart other beneficial medicinal qualities like high lipophilicity and better interaction with pathological biomolecules like nucleic acids (DNA) and proteins.

### 2.3. Preliminary Mechanistic Studies

Using coumarin derivative **11b** as a tool compound, we endeavoured to undertake introductory mechanistic investigation of the the presented compounds, targeting hemozoin inhibition (a mainstay antimalarial target) and DNA binding affinity. This was achieved by employing the detergent-mediated β-hematin (synthetic hemozoin) binding assay reported by Ncokazi and Egan for assessing hemozoin inhibitory effects of antimalarial compounds [60,61], and spectrophotometric DNA titration techniques (UV-Vis and fluorescence) adapted from the literature [62,63,64].

**Figure 3:**
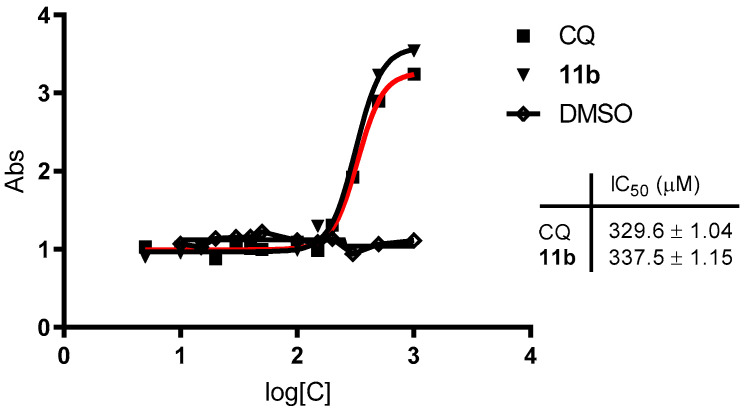
Hemozoin inhibition assay results of compound **11b** and CQ from the β-hematin binding assay. The margin of error is estimated in terms of the standard error (±SE).

The β-hematin inhibition was conducted as previously reported, employing varying concentrations of compound **11b** in DMSO alongside CQ as a control drug that was incubated with a fixed amount of β-hematin solution followed by the addition of NP-40 detergent to induce polymerisation [47]. The ability of compounds to inhibit hemozoin formation was monitored by measuring the absorbance of the unpolymerized haem complex at 405 nm and quantifying this response in terms of IC_50_ value (50% inhibitory concentration). The tool compound **11b** exhibited hemozoin inhibitory concentration of 337.5 ± 1.15 µM that was similar to that of CQ (IC_50_ = 329.6 ± 1.04 µM) (Figure 3). Considering that these values are comparable, although compound **11b** was significantly less potent than CQ against the 3D7 *P. falciparum* strain (Table 1), these results suggest that **11b** does not necessarily exert its plasmocidal activity by solely targeting hemozoin formation as the control drug. Effects such as cell and membrane penetrating ability of compound **11b** could be another reason to explain the discrepancies between its observed hemozoin inhibitory potential and antiplasmodial activity.

**Figure 4:**
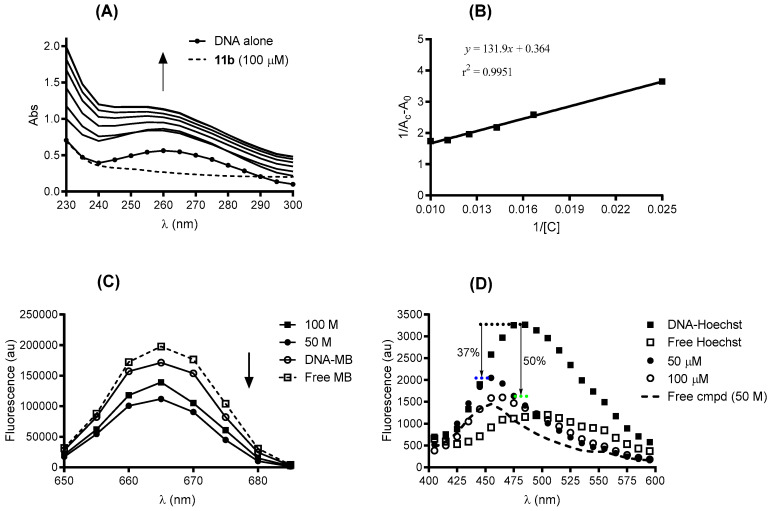
DNA binding affinity results of compound **11b** with calf thymus DNA. (**A**) UV-Vis DNA titration curve, (**B**) guest-host reciprocal plot of the UV-Vis DNA titration experiment, (**C**) competitive DNA intercalation assay using methylene blue (MB) and (**D**) Hoechst assay results for competitive DNA minor groove binding.

Next, compound **11b** was investigated for DNA binding affinity on mammalian calf thymus DNA employing UV-Vis DNA titration experiments. A constant amount of DNA was titrated with varying concentrations of compound **11b** and the absorbance measured at 260 nm, the maximal absorption wavelength of DNA, after an incubation period of 15 min at ambient temperature [63,64]. The interaction of the compound with DNA was confirmed by the dose-dependent hyperchromic shift of the DNA maximal absorption band accompanied by a slight hypsochromic (blue) shift of this band by approximately 4 nm (Figure 4A), suggesting external binding interactions via noncovalent associations [62,63,64]. The obtained data was fitted into the reciprocal guest-host equation (Equation (1)) to quantify the binding affinity of compound **11b** for DNA in terms of the binding constant (K_b_) (Figure 4B).

(1) $$\frac{1}{A_{c}-A_{0}}=\frac{1}{K_{b}\left(A_{\infty }-A_{0}\right)}\times \frac{1}{\left[C\right]}+\frac{1}{A_{\infty }-A_{0}}\;\;\;\;\;\;\;\;\;\;\;\;\;\;\;$$

where *A_c_* and *A*_0_ are the absorbance values of the DNA-incubated sample with different compound concentrations and unbound DNA sample at 260 nm, respectively, and *A_∞_* is the hypothetical DNA-compound complex. The K_b_ was determined as the ratio of the *y*-intercept to the slope of the equation generated by linear regression analysis of the plot of 1/(*A_c_* − *A*_0_) versus 1/[*C*] (Figure 4B). From the generated reciprocal guest-host linear equation (*y* = 131.9*x* + 0.364), a K_b_ of 2.77 × 10^3^ M^−1^ was obtained indicating moderate DNA binding affinity. This binding constant is comparative to the ones observed for the parental ferrocenyl 1,3−benzoxazines and aminocresols in the same order of magnitude [46,47,65].

Compound **11b** was further investigated for its mode of DNA binding employing a known DNA intercalator, i.e., methylene blue (MB) [66], and DNA minor groove binder, i.e., Hoechst 33342 [67,68], for either DNA intercalation or DNA minor groove binding, respectively, using previously reported methods (Figure 4C,D) [46,47]. Compound **11b** was unable to amplify fluorescence of the DNA-methylene blue sample (DNA-MB) at both tested concentrations (50 and 100 µM) (Figure 4C), which suggests its inability to displace DNA-bound (intercalated) methylene blue molecules from the DNA-MB complex since fluorescence of free MB molecules is what is monitored in the experiment, thus eliminating it as a DNA intercalator. However, in the assay for competitive minor groove binding with Hoechst 33342, the tool compound (**11b**) attenuated the intense, maximal fluorescence of the DNA-Hoechst complex at 425–525 nm by 37 and 50%, accompanied by blue shifts of 30 and 35 nm, for 50 and 100 µM tested concentrations, respectively (Figure 4D). In view of the fact that intense fluorescence only forms when DNA is bound by the Hoechst dye molecules in the minor groove [67,68], the interpretation of these experimental findings is that **11b** competes with the dye for DNA minor groove binding. Therefore, it is evident from these data that the mode of DNA interaction of compound **11b** is minor groove binding. 

## 3. Materials and Methods 

### 3.1. General Methods and Instrumentation

All the reagents and solvents used in this study were purchased from Merck (Johannesburg, South Africa) and used as received. In cases of reactions requiring anhydrous conditions the solvents were dried following established literature methods [69]. Formyl hydroxycoumarins (**9**, **14a**–**c**) and ferrocenyl amines (**10a**,**b**) were synthesised according to previously published procedures in the literature [13,51,52,53,54].

The progress of reactions was monitored by analytical thin layer chromatography (TLC) using Merck F254 silica gel plates coated on aluminium sheets and the plates were visualised under ultraviolet light (UV 254 and 366 nm). Crude compounds were purified by column chromatography with activated basic Brockmann alumina (Al_3_O_4_) as the stationary phase. The melting points were determined using Reichert melting point apparatus and were uncorrected. ^1^H and ^13^C NMR spectra were acquired on Bruker Biospin 300, 400 or 600 MHz spectrometers (Bruker, Ettlingen, Germany) and internally referenced using residual solvent signals of deuterated DMSO-*d*_6_: 2.50 ppm for 1H and 39.5 ppm for 13C NMR, or deuterated chloroform CDCl_3_: 7.26 ppm for ^1^H and 77.2 ppm for ^13^C NMR at ambient temperature. Representative 2D NMR spectra (COSY, HSQC and HMBC) were recorded on the 400 or 600 MHz spectrometers (Bruker, Ettlingen, Germany). The spectra were processed using MestReNova software. Chemical shifts of acquired spectra were reported in parts per million (ppm) and J-coupling constants measured in Hertz (Hz). Signal multiplicities were described by the following abbreviations: s = singlet, br s = broad singlet, d = doublet, t = triplet, q = quartet, and m = multiplet.

The high-resolution mass spectrometry (HRMS) data were acquired on Waters (Milford, MA, USA) Synapt G2 Mass Spectrometer (Central Analytical Facility, Stellenbosch University, Stellenbosch, South Africa) using electron impact (EI) ionization in the positive ionization mode, and the IR spectra were recorded on PerkinElmer Spectrum 100 FT-IR Spectrometer (Johannesburg, South Africa) in the mid-IR range (640–4000 cm^−1^). The HPLC purity of the compounds was determined using a reverse-phase Luna^®^ LC column (5 μM C18, 100 Å, 250 × 4.6 mm i.d.) on an Agilent 1100 Series HPLC instrument (Agilent, Santa Clara, CA, USA) equipped with a G1315B diode-array detector (DAD), G1311A quaternary pump, G1322A degasser and a G1328B manual injector. The compounds were run by isocratic elution in 10% MeOH/DCM (HPLC grade) for a total running time of 8 min. The calf thymus DNA was sourced from Thermo Fischer Scientific (Johannesburg, South Africa). The UV-Vis and fluorescent spectra for DNA titration experiments were recorded on a SpectraMax M3 microplate reader (v. 8.0.2, 2019, GraphPad Software, San Diego, CA 92108, USA).

### 3.2. Synthesis of Target Compounds

#### 3.2.1. General Procedure for Synthesis of Acyclic Derivatives **11a**,**b**

Formyl hydroxycoumarin **9** (1.0 eq.) was added to a solution of ferrocenyl amine **10a** or **10b** (1.0 eq.) in ethanol (15 mL) and the resulting mixture was refluxed for 4 h. The reaction mixture was cooled to room temperature following 4 h of reflux and sodium borohydride (2.0 eq.) was cautiously added. The reaction was then stirred at room temperature for 15 min and refluxed for another 15 min after which it was allowed to cool to room temperature. The product was extracted into the aqueous phase with 2N HCl solution (2 × 25 mL) and washed with EtOAc (25 mL). The collected aqueous layers were basified with 1N NaOH solution (pH 10) and the product extracted with DCM (2 × 25 mL), dried (Na_2_SO_4_) and solvent removed in vacuo to afford the title compound. Column chromatography on basic alumina was employed to purify the compounds where necessary using gradient elution (DCM→1:9 MeOH/DCM).

*8-((Ferrocenemethyl)aminomethyl)-7-hydroxycoumarin* (**11a**). Light brown semi-solid. Yield: 235.2 mg (86%). ^1^H NMR (600 MHz, DMSO-*d*_6_) *δ* 7.83–7.81 (m, 2H, H_5_, H_7_), 7.34 (t, *J* = 7.4 Hz, 1H, H_6_), 7.10–7.03 (m, 2H, H_6_, H_8_), 4.16 (s, 2H, FcH), 4.13 (s, 2H, FcH), 4.10 (s, 5H, FcH), 4.02 (s, 2H, H_3’_), 3.55 (s, 2H, H_1’_); ^13^C NMR (150 MHz, DMSO-*d_6_*) *δ* 173.1, 165.0, 153.9, 129.4, 124.7, 123.6, 121.5, 115.5, 95.1, 88.9, 68.2 (5C), 67.4 (2C), 66.8 (2C), 46.9, 44.2; IR (ATR, cm^−1^) ν = 3080 (O-H), 2914 (N-H); HRMS (ESI^+^) *m*/*z* calcd for C_21_H_20_FeNO_3_ [M + H]^+^: 390.0793, Found 390.0782; HPLC purity > 99% (*t*_R_ = 2.47 min).

*8-((N,N-Dimethylamino)methyl)ferrocenemethyl)-7-hydroxycoumarin* (**11b**). Light brown semi-solid. Yield: 121.6 mg (56%). ^1^H NMR (600 MHz, CDCl_3_) *δ* 7.59 (d, *J* = 9.3 Hz, 1H, H_3_), 7.23 (d, *J* = 8.4 Hz, 1H, H_6_), 6.72 (d, *J* = 8.4 Hz, 1H, H_5_), 6.10 (d, *J* = 9.3 Hz, 1H, H_4_), 4.22 (br s, 1H, FcH), 4.18 (br s, 1H, FcH), 4.13–4.12 (m, 2H, H_8’_), 4.09 (br s, 1H, FcH), 4.04 (s, 5H, FcH), 3.91 (d, *J* = 13.2 Hz, 1H, H_2’a_), 3.79 (d, *J* = 12.7 Hz, 1H, H_1’a_), 3.42 (d, *J* = 13.2 Hz, 1H, H_2’b_), 2.80 (d, *J* = 12.6 Hz, 1H, H_1’b_), 2.15 (s, 6H, NMe_2_); ^13^C NMR (150 MHz, CDCl_3_) *δ* 161.9, 153.6, 144.7, 128.1, 115.1, 110.2, 110.1, 107.4, 100.1, 84.2, 82.6, 69.9, 69.5, 69.2 (5C), 69.0, 58.4, 46.4, 44.9 (2C), 44.6; IR (ATR, cm^−1^) ν = 3085 (O-H), 2918 (N-H); HRMS (ESI^+^) *m*/*z* calcd for C_24_H_27_FeN_2_O_3_ [M + H]^+^: 447.1371, Found 447.1371; HPLC purity > 99% (*t*_R_ = 2.46 min).

#### 3.2.2. General Procedure for Synthesis of Cyclic Coumarin-1,3-oxazin-2-one Derivatives **12a**,**b**

CDI (1.2 eq.) was added to a solution of a relevant acyclic ferrocenyl aminohydroxycoumarin (**11a** or **11b**) (1.0 eq.) in DCM (15 mL). The resultant reaction mixture was stirred at room temperature for 1–2 h. After completion of the reaction (TLC), unreacted CDI was quenched by the addition of 0.5N NaOH solution (15 mL) and the product extracted with DCM (2 × 25 mL). The combined organic layers were washed with distilled water (25 mL), dried (Na_2_SO_4_) and the solvent was removed under reduced pressure to furnish the desired carbamate products **12a**,**b** in high purity.

*9-Ferrocenemethyl-9,10-dihydro-2H,8H-chromeno[8,7-e][1,3]oxazine-2,8-dione* (**12a**). Light brown semi-solid. Yield: 146.0 mg (96%). M.p.: 145.4–148.0 °C. ^1^H NMR (400 MHz, DMSO-*d*_6_/Acetone-*d*_6_) *δ* 8.03 (d, *J* = 9.3 Hz, 1H, H_3_), 7.66 (d, *J* = 8.4 Hz, 1H, H_6_), 7.01 (d, *J* = 8.2 Hz, 1H, H_5_), 6.41 (d, *J* = 9.4 Hz, 1H, H_4_), 4.57 (s, 2H, H_10_), 4.47 (s, 2H, H_1’_), 4.40 (s, 2H, FcH), 4.23 (s, 5H, FcH), 4.19 (s, 2H, FcH); ^13^C NMR (100 MHz, DMSO-*d*_6_/Acetone-*d*_6_) *δ* 159.3, 151.6, 150.1, 148.2, 144.1, 128.7, 114.8, 114.2, 112.0, 105.8, 81.4, 69.4, 68.5, 68.2, 47.9, 42.0; IR (ATR, cm^−1^) ν = 1716 (C=O); HRMS (ESI^+^) *m*/*z* calcd for C_21_H_20_FeNO_3_ [M − CO + H_3_]^+^: 390.0787, Found 390.0781; HPLC purity > 99% (*t*_R_ = 2.50 min).

*9-(2-((N,N-Dimethylamino)methyl)ferrocenemethyl)-9,10-dihydro-2H,8H-chromeno[8,7-e][1,3]oxazine-2,8-dione* (**12b**). Light yellow solid. Yield: 62.2 mg (90%). M.p.: 105.3–108.6 °C. ^1^H NMR (400 MHz, CDCl_3_) *δ* 7.63 (d, *J* = 9.6 Hz, 1H, H_3_), 7.34 (d, *J* = 8.5 Hz, 1H, H_6_), 6.91 (d, *J* = 8.5 Hz, 1H, H_5_), 6.31 (d, *J* = 9.6 Hz, 1H, H_4_), 4.73–4.68 (m, 2H, H_10_), 4.55–4.48 (m, 2H, H_2’_), 4.39 (br s, 1H, FcH), 4.29 (br s, 1H, FcH), 4.16 (t, *J* = 2.4 Hz, 1H, FcH), 4.13 (s, 5H, FcH), 3.83–3.70 (m, 1H, H_1’a_), 3.15–3.03 (m, 1H, H_1’b_), 2.19 (s, 6H, NMe_2_); ^13^C NMR (100 MHz, CDCl_3_) *δ* 160.0, 152.3, 150.5, 149.4, 143.3, 127.9, 114.9, 114.8, 112.7, 106.5, 80.9 (2C), 71.9, 71.2, 69.5 (5C), 67.8, 57.2, 46.9, 44.4 (2C), 41.9; IR (ATR, cm^−1^) ν = 1720 (C=O); HRMS (ESI^+^) *m*/*z* calcd for C_24_H_25_FeN_2_O_4_ [M + H]^+^: 473.1164, Found 473.1159;); HPLC purity > 99% (*t*_R_ = 3.43 min).

#### 3.2.3. Synthesis of 9-(2-((*N*,*N*-Dimethylamino)methyl)ferrocenemethyl)-9,10-dihydrochromeno[8,7-e][1,3]oxazin-2(8H)-one (**13**)

Aminohydroxycoumarin **11b** (1.0 eq.) in CHCl_3_ (20 mL) was refluxed with paraformaldehyde (1.0 eq.) for 12 h, after which the reaction was cooled to room temperature. The reaction mixture was diluted with CHCl_3_ (50 mL) and successively washed with 1N NaOH solution (2 × 25 mL) and distilled water (25 mL). The organic layer was dried (Na_2_SO_4_) and the solvent removed in vacuo to afford the target compound in high purity as a light yellow semi-solid. Yield: 94.3 mg (68%). ^1^H NMR (600 MHz, CDCl_3_) *δ* 7.62 (d, *J* = 9.4 Hz, 1H, H_3_), 7.24 (s, 1H, H_6_), 6.76 (d, *J* = 8.5 Hz, 1H, H_5_), 6.22 (d, *J* = 9.4 Hz, 1H, H_4_), 4.96 (s, 2H, H_8_), 4.26 (br s, 1H, H, H_10a_), 4.22–4.21 (m, 2H, FcH, H_8b_), 4.12 (t, *J* = 2.5 Hz, 1H, FcH), 4.06 (s, 1H, FcH), 4.01 (s, 5H, FcH), 3.77 (d, *J* = 13.3 Hz, 1H, H_2’a_), 3.73 (d, *J* = 13.3 Hz, 1H, H_2’b_), 3.31–3.27 (m, 1H, H_1’a_), 3.10 (d, *J* = 12.9 Hz, 1H, H_2’b_), 2.07 (s, 6H, NMe_2_); ^13^C NMR (150 MHz, CDCl_3_) *δ* 161.3, 158.0, 152.8, 144.1, 126.5, 113.7, 112.6, 112.2, 108.7, 84.4, 83.3, 82.9, 70.8, 70.3, 69.4 (5C), 67.4, 57.5, 49.8, 45.3 (2C), 44.6; IR (ATR, cm^−1^) ν = 1238 (C-O), 1116 (C-N); HRMS (ESI^+^) *m*/*z* calcd for C_25_H_27_FeN_2_O_3_ [M + H]^+^: 459.1366, Found 459.1363; HPLC purity > 99% (*t*_R_ = 3.40 min).

#### 3.2.4. Synthesis of Acyclic Ferrocenyl Aminocoumarin-4-ols **15a**–**d**

Compounds **15a**–**d** were prepared from substituted 3-formyl-4-hydroxycoumarins **14a**–**c** and ferrocenyl amines **10a**,**b** following the reaction conditions described for synthesis acyclic derivatives **11a**,**b**.

*3-((Ferrocenemethyl)aminomethyl)-coumarin-4-ol* (**15a**). Light brown semi-solid: 235.2 mg (86%). ^1^H NMR (600 MHz, DMSO-*d*_6_) *δ* 7.83–7.81 (m, 2H, H_5_, H_7_), 7.34 (t, *J* = 7.4 Hz, 1H, H_6_), 7.10–7.03 (m, 2H, H_5_, H_8_), 4.16 (s, 2H, FcH), 4.13 (s, 2H, FcH), 4.10 (s, 5H, FcH), 4.02 (s, 2H, H_1’_), 3.55 (s, 2H, H_1’_); ^13^C NMR (150 MHz, DMSO-*d_6_*) *δ* 173.1, 165.0, 153.9, 129.4, 124.7, 123.6, 121.5, 115.5, 95.1, 88.9, 68.2 (5C), 67.4 (2C), 66.8 (2C), 46.9, 44.2; IR (ATR, cm^−1^) ν = 3083 (O-H), 2915 (N-H); HRMS (ESI^+^) *m*/*z* calcd for C_21_H_20_FeNO_3_ [M + H]^+^: 390.0793, Found 390.0779; HPLC purity > 91% (*t*_R_ = 2.47 min).

*3-((2-((N,N-Dimethylamino)methyl)ferrocenemethyl)aminomethyl)-coumarin-4-ol* (**15b**). Brown semi-solid: 350.2 mg (68%). ^1^H NMR (600 MHz, CDCl_3_ *δ* 7.83 (dd, *J* = 7.7, 1.1 Hz, 1H, H_5_), 7.40 (td, *J* = 7.7, 1.4 Hz, 1H, H_7_), 7.17 (d, *J* = 7.8 Hz, 1H, H_8_), 7.14 (t, *J* = 7.5 Hz, 1H, H_6_), 4.34 (s, 1H, FcH), 4.19 (br s, 1H, FcH), 4.20–4.17 (m, 2H, H_3’a_, FcH), 4.13 (t, *J* = 2.4 Hz, 1H, FcH), 4.11 (s, 5H, FcH), 4.09 (d, *J* = 8.8 Hz, 2H, H_2’_), 3.90–3.89 (m, 2H, H_1’a_, H_3’b_), 2.88 (d, *J* = 13.0 Hz, 1H, H_1’b_), 2.16 (s, 6H, NMe_2_); ^13^C NMR (150 MHz, CDCl_3_) *δ* 176.1, 165.8, 154.4, 131.1, 124.5, 122.8, 122.0, 116.6, 100.1, 88.8, 84.1, 72.0 (2C), 69.7 (5C), 67.3, 58.1, 45.7, 44.2, 43.7 (2C); IR (ATR, cm^−1^) ν = 2820 (O-H), 2774 (N-H); HRMS (ESI^+^) *m*/*z* calcd for C_24_H_27_FeN_2_O_3_ [M + H]^+^: 447.1371, Found 447.1371; HPLC purity > 96% (*t*_R_ = 2.46 min).

*3-((2-((N,N-Dimethylamino)methyl)ferrocenemethyl)aminomethyl)-6-fluoro-4-hydroxycoumarin* (**15c**). Light brown semi-solid. 109.0 mg (64%). ^1^H NMR (600 MHz, CDCl_3_) *δ* 7.48 (dd, *J* = 8.6, 2.9 Hz, 1H, H_5_), 7.14–7.09 (m, 2H, H_7_, H_8_), 4.33 (br s, 1H, FcH), 4.20 (br s, 1H, FcH), 4.18 (d, *J* = 13.4 Hz, 1H, H_2’a_), 4.13 (t, *J* = 2.4 Hz, 1H, FcH), 4.11 (s, 5H, FcH), 4.07 (d, *J* = 13.0 Hz, 2H, H_3’_), 3.87 (d, *J* = 13.1 Hz, 2H, H_1’a_, H_2’b_), 2.89 (d, *J* = 13.0 Hz, 1H, H_1’b_), 2.16 (s, 6H, NMe_2_); ^13^C NMR (150 MHz, CDCl_3_) *δ* 174.9, 165.8, 159.3, 157.7, 150.3, 123.2 (d, *J* = 7.9 Hz), 118.3 (d, *J* = 24.8 Hz), 118.0 (d, *J* = 7.9 Hz), 109.9 (d, *J* = 23.8 Hz), 89.2, 84.1, 72.0, 71.9, 69.6 (5C), 67.3, 58.1, 46.0, 44.2 (2C), 43.5; IR (ATR, cm^−1^) ν = 3076 (O-H), 2915 (N-H); HRMS (ESI^+^) *m*/*z* calcd for C_24_H_26_FFeN_2_O_3_ [M + H]^+^: 465.1279, Found 465.1279; HPLC purity > 99% (*t*_R_ = 2.48 min).

*3-((2-((N,N-Dimethylamino)methyl)ferrocenemethyl)aminomethyl)-6-chloro-4-hydroxycoumarin* (**15d**). Light brown semi-solid. Yield: 174.4 mg (66%). ^1^H NMR (600 MHz, CDCl_3_) *δ* 7.75 (d, *J* = 2.5 Hz, 1H, H_5_), 7.31 (dd, *J* = 8.7, 2.6 Hz, 1H, H_7_), 7.09 (d, *J* = 8.7 Hz, 1H, H_4_), 4.31 (br s, 1H, FcH), 4.21 (d, *J* = 13.3 Hz, 1H_3’a_), 4.19 (br s, 1H, FcH), 4.11 (d, *J* = 4.7 Hz, 6H, FcH), 4.06–4.01 (m, 2H, H_2’_), 3.88 (d, *J* = 13.0 Hz, 1H, H_1’a_), 3.85 (d, *J* = 13.3 Hz, 1H, H_3’b_), 2.87 (d, *J* = 13.0 Hz, 1H, H_1’b_), 2.15 (s, 6H, NMe_2_); ^13^C NMR (150 MHz, CDCl_3_) *δ* 174.6, 165.5, 152.7, 130.9, 128.2, 124.2, 123.4, 118.1, 100.1, 89.4, 84.1, 72.0, 71.9, 69.6 (5C), 67.3, 58.1, 46.0, 44.2 (2C), 43.3; IR (ATR, cm^−1^) ν = 3070 (O-H), 2923 (N-H); HRMS (ESI^+^) *m*/*z* calcd for C_24_H_26_ClFeN_2_O_3_ [M + H]^+^: 481.0976, Found 481.1568; HPLC purity > 99% (*t*_R_ = 2.47 min).

#### 3.2.5. Synthesis of Ferrocenyl Coumarin-1,3-oxazin-2-one Derivatives **16a**–**d**

Compounds **16a**–**d** were synthesized according to the general procedure employed for the synthesis of coumarin-1,3-oxazin-2-one derivatives **12a**,**b** from aminocoumarin-4-ols **15a**–**d**.

*3-Ferrocenemethyl-3,4-dihydro-2H,5H-chromeno[3,4-e][1,3]oxazine-2,5-dione* (**16a**). Light brown semi-solid. Yield: 41.0 mg (68%). ^1^H NMR (600 MHz, CDCl_3_) *δ* 7.78 (dd, *J* = 8.1, 1.4 Hz, 1H, H_7_), 7.51 (td, *J =* 8.4, 1.4 Hz, 1H, H_8_), 7.35–7.33 (m, 2H, H_9_, H_10_), 4.40 (s, 2H, H_4_), 4.26 (br s, 2H, FcH), 4.12 (br s, 9H, FcH, H_1’_); ^13^C NMR (150 MHz, CDCl_3_) *δ* 159.8, 155.9, 153.2, 147.1, 133.1, 124.9, 122.8, 117.0, 113.0, 98.2, 80.1, 70.0 (2C), 69.2 (2C), 68.9 (5C), 49.3, 43.3; IR (ATR, cm^−1^) ν = 1738 (C=O); HRMS (ESI^+^) *m*/*z* calcd for C_22_H_18_FeNO_4_ [M + H]^+^: 416.0586, Found 416.0586; HPLC purity > 98% (*t*_R_ = 3.03 min).

*3-(2-((N,N-Dimethylamino)methyl)ferrocenemethyl)-3,4-dihydro-2H,5H-chromeno[3,4-e][1,3]oxazine-2,5-dione* (**16b**). Light brown semi-solid. Yield: 37.3 mg (75%). ^1^H NMR (600 MHz, CDCl_3_) *δ* 7.88 (d, *J* = 7.8 Hz, 1H, H_7_), 7.58 (t, *J* = 7.6 Hz, 1H, H_8_), 7.33–7.33 (m, 2H, H_9_, H_10_), 4.81 (d, *J* = 14.5 Hz, 1H, H_4a_), 4.38 (d, *J* = 14.5 Hz, 1H, H_4b_), 4.33–4.31 (m, 2H, H_2’a_, FcH), 4.28 (s, 1H, FcH), 4.21 (br s, 1H, FcH), 4.14–4.13 (m, 6H, H_2’b_, FcH), 3.66 (d, *J* = 12.4 Hz, 1H, H_1’a_), 2.90 (br s, 1H, H_1’b_), 2.10 (s, 6H, NMe_2_); ^13^C NMR (150 MHz, CDCl_3_) *δ* 159.8, 155.6, 153.2, 147.6, 132.8, 124.8, 122.7, 117.0, 113.1, 98.8, 88.5, 80.5, 72.0, 71.3, 69.5 (5C), 67.5, 57.5, 47.2 (2C), 44.9, 42.5; IR (ATR, cm^−1^) ν = 1752 (C=O); HRMS (ESI^+^) *m*/*z* calcd for C_24_H_27_ClFeN_2_O_3_Na [M − CO + NaCl + H_3_]^+^: 505.0952, Found 505.1424; HPLC purity > 91% (*t*_R_ = 2.43 min).

*3-(2-((N,N-Dimethylamino)methyl)ferrocenemethyl)-9-fluoro-3,4-dihydro-2H,5H-chromeno[3,4-e][1,3]oxazine-2,5-dione* (**16c**). Light brown semi-solid. Yield: 16.6 mg (36%). ^1^H NMR (600 MHz, CDCl_3_) *δ* 7.56 (dd, *J* = 8.0, 2.8 Hz, 1H, H_10_), 7.33–729 (m, 2H, H_7_, H_18_), 4.79 (d, *J* = 14.5 Hz, 1H, H_4a_), 4.39 (d, *J* = 14.5 Hz, 1H, H_4b_), 4.34–4.29 (m, 2H, H_2’a_, FcH), 4.18 (s, 1H, FcH), 4.15–4.09 (m, 7H, H_2’b_, FcH), 3.64 (d, *J* = 12.6 Hz, 1H, H_1’a_), 2.84 (d, *J* = 12.6 Hz, 1H, H_1’b_), 2.07 (s, 6H); ^13^C NMR (150 MHz, CDCl_3_) *δ* 159.5, 158.2, 154.8, 149.3, 147.2, 120.4 (d, *J* = 24.6 Hz), 118.7 (d, *J* = 8.3 Hz), 114.1, 108.6 (d, *J* = 25.9 Hz), 99.8, 84.9, 80.3, 72.1, 71.3, 69.5 (5C), 67.4, 57.6, 47.3, 45.0 (2C), 42.5; IR (ATR, cm^−1^) ν = 1717 (C=O); HRMS (ESI^+^) *m*/*z* calcd for C_24_H_26_ClFFeN_2_O_3_Na [M − CO + NaCl + H_3_]^+^: 523.1338, Found 523.1338; HPLC purity > 91% (*t*_R_ = 2.60 min).

*3-(2-((N,N-Dimethylamino)methyl)ferrocenemethyl)-9-chloro-3,4-dihydro-2H,5H-chromeno[3,4-e][1,3]oxazine-2,5-dione* (**16d**). Light brown semi-solid. Yield: 17.3 mg (49%). ^1^H NMR (600 MHz, CDCl_3_) *δ* 8.06 (d, *J* = 2.4 Hz, 1H, H_10_), 7.71 (dd, *J* = 8.8, 2.4 Hz, 1H, H_8_), 7.47 (d, *J* = 8.9 Hz, 1H, H_7_), 4.98 (d, *J* = 14.5 Hz, 1H, H_4a_), 4.59 (d, *J* = 14.5 Hz, 1H, H_4b_), 4.51–4.49 (m, 2H, FcH, H_2’a_), 4.38 (br s, 1H, FcH), 4.33–4.32 (m, 7H, FcH, H_2’b_), 3.83 (d, *J* = 12.5 Hz, 1H, H_1’a_), 3.03 (d, *J* = 12.6 Hz, 1H, H_1’b_), 2.27 (s, 6H, NMe_2_); ^13^C NMR (150 MHz, CDCl_3_) δ 159.2, 154.5, 151.4, 147.1, 132.9, 130.5, 122.3, 118.4, 114.2, 99.8, 84.9, 80.3, 72.1, 71.3, 69.5 (5C), 67.4, 57.6, 47.2, 45.0 (2C), 42.4; IR (ATR, cm^−1^) ν = 1717 (C=O); HRMS (ESI^+^) *m*/*z* calcd for C_24_H_26_Cl_2_FeN_2_O_3_Na [M − CO + NaCl + H_3_]^+^: 539.1057, Found 539.1057; HPLC purity > 99% (*t*_R_ = 2.58 min).

#### 3.2.6. Synthesis of 7-(2-((*N*,*N*-Dimethylamino)methyl)ferrocenemethyl)-7,8-dihydro-6H-[1,3]*dioxolo[4’,5’:4,5]benzo[1,2-e][1,3]oxazine* (**19**)

Compound **19** was synthesized from sesamol **18** (1.0 eq) and paraformaldehyde (2.0 eq.) following the procedure described for the synthesis of coumarin-oxazine **13**. Brown semi-solid. Yield: 135.5 mg (85%). ^1^H NMR (400 MHz, CDCl_3_) *δ* 6.39 (s, 1H, H_9_), 6.38 (s, 1H, H_4_), 5.87 (s, 2H, H_2_), 4.85–4.77 (m, 2H, H_6_), 4.25 (br s, 1H, FcH), 4.22 (br s, 1H, FcH), 4.12–4.11 (m, 1H, FcH), 4.02 (s, 5H, FcH), 3.93 (br s, 2H, H_8_), 3.73 (s, 2H, H_2’_), 3.30 (d, *J* = 12.9 Hz, 1H, H_1’a_), 3.19 (d, *J* = 12.9 Hz, 1H, H_1’b_), 2.16 (s, 6H, NMe_2_); ^13^C NMR (100 MHz, CDCl_3_) *δ* 148.8, 146.6, 141.5, 111.7, 106.9, 100.8, 98.5, 84.2, 83.9, 82.4, 70.7, 70.3, 69.4 (5C), 67.2, 57.4, 49.3, 49.3, 45.4 (2C); IR (ATR, cm^−1^) ν = 1241 (C-O), 1141 (C-N), HRMS (ESI^+^) *m*/*z* calcd for C_23_H_27_FeN_2_O_3_ [M + H]^+^: 435.1366, Found 435.1370; HPLC purity > 99% (*t*_R_ = 2.40 min).

### 3.3. Biological Evaluation Assays

#### 3.3.1. D7 *P. falciparum* Antiplasomidal Evaluation

The 3D7 *P. falciparum* parasites were grown in a RPMI1640 medium containing 25 mM HEPES (Lonza, Switzerland), 2–4% (*v*/*v*) human erythrocytes, 0.5% (*w*/*v*) Albumax II (Thermo Fisher Scientific, Waltham, MA, USA), 22 mM glucose, 0.65 mM hypoxanthine and 0.05 mg/mL gentamicin and kept at 37 °C under a 5% CO_2_, 5% O_2_ and 90% N_2_ atmosphere. The cultures were transferred to 96-well plates and then treated with serial dilutions, in DMSO, of the test compounds and chloroquine as a positive control. Following a 48-h incubation period under these conditions, the plasmocidal activity of each compound was quantified in terms of IC_50_ values according to previously reported procedures using the plasmodium lactate dehydrogenase (pLDH) enzyme assay [47,70].

#### 3.3.2. *T. b. Brucei* 427 Antitrypanosomal Assay

Trypomastigotes of the *T. b. brucei* 427 strain were cultured in an IMDM medium (Lonza, Switzerland) supplemented with 10% fetal serum, HMI-9 supplement, hypoxanthine and penicillin/streptomycin in a 10% CO_2_ incubator maintained at 37 °C. The parasites were seeded into 96-well plates and incubated with varying concentrations of the test compounds and control drug (pentamidine) for a period of 24 h after which resazurin was added to a final concentration of 50 µM. Following a further 24-h incubation under the same conditions, the antitrypanosomal activity of the compounds was quantified in terms of the IC_50_ values according to the previously described procedure [46].

#### 3.3.3. Antiproliferative Assay against the HCC70 Triple-Negative Breast Cancer Cell Line

The compounds were evaluated for anticancer efficacy against HCC70 triple-negative breast cancer cells (ATCC^®^ CRL-2315™) that were cultured and screened according to the methods used in the previous studies [46,47].

#### 3.3.4. β-Hematin Binding Assay for Hemozoin Inhibition

Varying concentrations of the tool compound (**11b**) and chloroquine dissolved in DMSO were mixed with 100 µL of 220 µM hematin solution (prepared from hemin from porcine in DMSO and 1.0 M acetate buffer, pH 4.8) in a 96-well plate. Hemozoin formation was induced by adding 20 µL of 30 µM NP-40 detergent followed by 6-h incubation of the plate at 37 °C with gentle shaking. Following incubation, a solution of 50% pyridine (*v*/*v*), 20% acetone (*v*/*v*), 10% distilled water and 20% 200 mM HEPES buffer (pH 7.4) were added to each well to analyse hemozoin formation by the pyridine-ferrichrome method [60,61]. Dispersion of the formed hemozoin solid was facilitated by adding 50 µL acetone and shaking the plate for 10 min at 37 °C after which absorbance was measured at 405 nm in each well. The recorded absorbance was plotted against the logarithm of the corresponding concentration in each well to quantify hemozoin inhibition activity (IC_50_ values) by non-linear regression analysis in GraphPad Prism (v. 8.0.2, 2019, GraphPad Software, San Diego, CA 92108, USA).

#### 3.3.5. UV-Vis DNA Titration Experiment for DNA Binding

A fixed concentration of calf thymus DNA (70 ng/µL) in a 96-well plate was treated with different concentrations of compound **11b** (range: 1–100 µM) prepared from a 20 mM stock solution (in DMSO) with milli-Q water and incubated at 37 °C for 15 min. This was followed by recording the absorbance of the samples in each well from 230–290 nm on a SpectraMax M3 microplate plate reader (Molecular Devices, San Jose, CA, USA). Absorbance of the 100 μM compound sample (without DNA) as well as unbound DNA were similarly treated. The binding affinity of the tool compound (**11b**) was determined by plotting concentration-dependent response curves as spectra showing absorbance over the tested wavelength range. DNA binding affinity was quantified as the binding constant (K_b_) determined as a ratio of the *y*-intercept to slope of the guest-host reciprocal plot (Equation (1)) depicting the relationship between maximal absorbance (around 260 nm) in each well to the corresponding compound concentration.

#### 3.3.6. Methylene Blue and Hoechst 33342 DNA Binding Assay for Competitive Intercalation or Minor Groove Binding

Concentrations of compound **11b** (0, 50 and 100 µM), prepared as described above, were placed with calf thymus DNA (70 ng/µL) in a 96-well plate after which a solution of methylene blue (1.5 µg/mL) or Hoechst 33342 dye (1.0 µg/mL) in distilled water were added to each well. The samples were kept in the dark for 15 min at ambient temperature and the fluorescence spectra were recorded (*λ*_ex_ = 665 nm, *λ*_em_ 650–750 nm for methylene blue; *λ*_ex_ = 350 nm, *λ*_em_ = 400–600 nm for Hoechst 33342) and the results interpreted according to each dye’s DNA binding mode.

## 4. Conclusions

In the current study, the hybridisation strategy was employed to assemble bioactive chemical moieties, ferrocene, coumarin and oxazine, into a focused series of ferrocenyl coumarin-1,3-oxazine derivatives with the aim of expanding on the previous work on related ferrocenyl benzoxazines and α-aminocresols by investigating the pharmacological effects of annulling coumarin to the phenyl unit of these compounds. Biological evaluation of these coumarin-annulated derivatives on the CQS 3D7 *P. falciparum* strain and *T. b. brucei* 427 trypomastigotes shows that the introduction of the coumarin unit is tolerated for biological activity of this class of compounds mostly in the low micromolar range, albeit lower potency is also observed. This structural modification proved limiting for anticancer activity against the HCC70 triple-negative breast cancer cell line. To gain insight into the plausible mechanistic action of the compounds, compound **11b** showing promising potency against both parasitic strains was assessed for potential hemozoin inhibition and DNA interaction and was found to act on both targets, suggesting that this class of compounds likely interacts with these biomolecules to exhibit biological activity similar to the parent benzoxazine and aminocresol counterparts. Overall, the current study sheds more light into the enriched SAR model of ferrocenyl 1,3-benzoxazines and aminocresols and paves the way for further structural optimisation of these compounds to produce better performing analogues as antiparasitic agents for future investigation.

## Figures and Tables

**Table 1:** Antiplasmodial and antitrypanosomal activities of target compounds against 3D7 *P. falciparum* trophozoites and nagana *T. b. brucei* 427 trypomastigotes.

Compound	IC_50_ (µM)	
	3D7 *P. falciparum*	*T. b. brucei* 427
**11a**	9.1	n.a. ^a^
**11b**	1.7	9.4
**12a**	n.a.	n.a.
**12b**	7.3	n.a.
**13**	1.0	n.d. ^b^
**15a**	n.a.	n.a.
**15b**	n.a.	n.a.
**15c**	8.76	19.6
**15d**	5.7	5.0
**16a**	13.2	n.a.
**16b**	n.a.	n.a.
**16c**	12.55	n.a.
**16d**	n.a.	n.d.
**19**	4.78	n.d.
**20a ^c^**	3.49	n.a.
**20b ^c^**	4.12	0.87
**CQ**	0.03	–
**Pentamidine**	–	0.014

^a^ not determined; ^b^ not determined; ^c^ biological data was adapted from a previously reported study [46].

## Data Availability

The data provided in this study are available in the article and supplementary material file submitted.

## Supplementary Materials

The following are available online, the NMR spectral data and infor-mation pertaining to the HeLa cell evaluation bioassay, such as Figure S1: Results of preliminary cytotoxicity evaluation assay showing percentage viability of HeLa cells plotted in parallel with percentage growth of 3D7 P. falciparum parasites treated with 20 µM fixed concentration of the test compounds, can be accessed via the online version of this paper.
