# Supplementary material for: Coumarin-Annulated Ferrocenyl 1,3-Oxazine Derivatives Possessing In Vitro Antimalarial and Antitrypanosomal Potency
Source: Molecules. 2021 Mar 2;26(5):1333. doi: 10.3390/molecules26051333 (PMC7958634; doi:10.3390/molecules26051333)
Supplement: Supplementary file 1 [file molecules-26-01333-s001.pdf]

Supporting Information:

Coumarin-annulated ferrocenyl 1,3-oxazine derivatives possessing *in vitro* antimalarial and antitrypanosomal potency

Mziyanda Mbaba [1], Laura M.K. Dingle [2,3], Ayanda I. Zulu [1], Dustin Laming [2,4], Tarryn Swart [2,4], Jo-Anne de La Mare [2,3,4], Heinrich C. Hoppe [2,4], Adrienne L. Edkins [2,3,4] and Setshaba D. Khanye [1,4,5,*]

[1] Department of Chemistry, Faculty of Science, Rhodes University, Makhanda, 6140; mziyanda.mbaba@uct.ac.za (M.M.); azulu50@gmail.com (A.I.Z.); s.khanye@ru.ac.za (S.D.K.)

[2] Department of Biochemistry and Microbiology, Faculty of Science, Rhodes University, Makhanda, 6140; dustinlaming89@gmail.com (D.L.); g10s2905@campus.ru.ac.za (T.S.); g11d1612@campus.ru.ac.za (L.M.K.D.); j.delamare@ru.ac.za (J.DLM.); h.hoppe@ru.ac.za (H.C.H.); a.edkins@ru.ac.za (A.L.E.)

[3] Biomedical Biotechnology Research Unit, Rhodes University, Makhanda, 6140

[4] Centre for Chemico- and Biomedicinal Research, Rhodes University, Makhanda, 6140

[5] Division of Pharmaceutical Chemistry, Faculty of Pharmacy, Rhodes University, Makhanda, 6140

[*] Correspondence: s.khanye@ru.ac.za; Tel.: +27-46-603-8397

Table of contents

General cytotoxicity evaluation assay against the HeLa cell line (3)

Figure S1 (3)

NMR spectral data of investigated compounds (4)

General cytotoxicity evaluation assay against the HeLa cell line

HeLa cells (Cellonex, South Africa) were cultured in in Dulbecco's modified Eagle's medium (Lonza, Switzerland) containing 10% foetal calf serum and antibiotics (penicillin, streptomycin and amphotericin B) in a 5% CO_2 incubator kept at 37°C. Equal densities of the cells were seeded in a 96-well plate and incubated 24 hours. The seeded cells in the wells were treated with a fixed concentration (20 μM) of test compound and further incubated under the same conditions for 48 hours. The viability of cells in each treated well was measured on a SpectraMax M3 microplate reader employing the resazurin procedure as previously described [1].

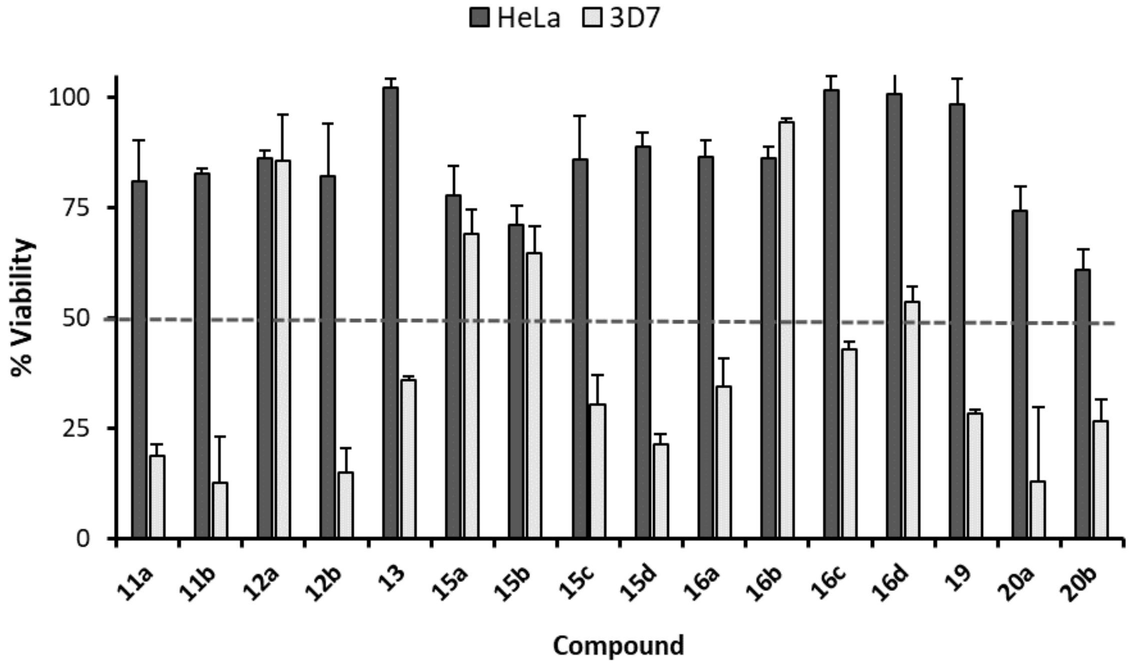

Figure S1. Results of preliminary cytotoxicity evaluation assay showing percentage viability of HeLa cells plotted in parallel with percentage growth of 3D7 *P. falciparum* parasites treated with 20 μM fixed concentration of the test compounds.

^{1}H NMR spectrum of compound **11a**

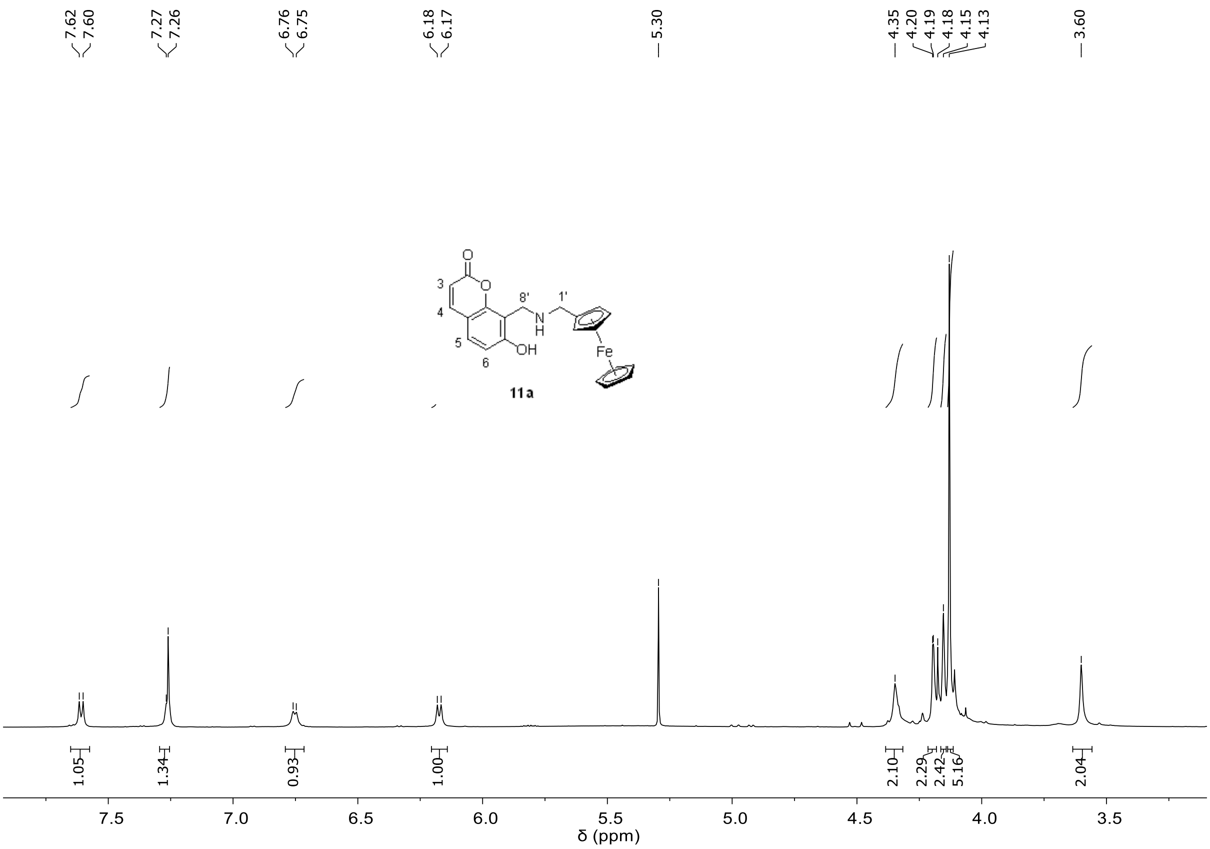

^{13}C NMR spectrum of compound **11a**

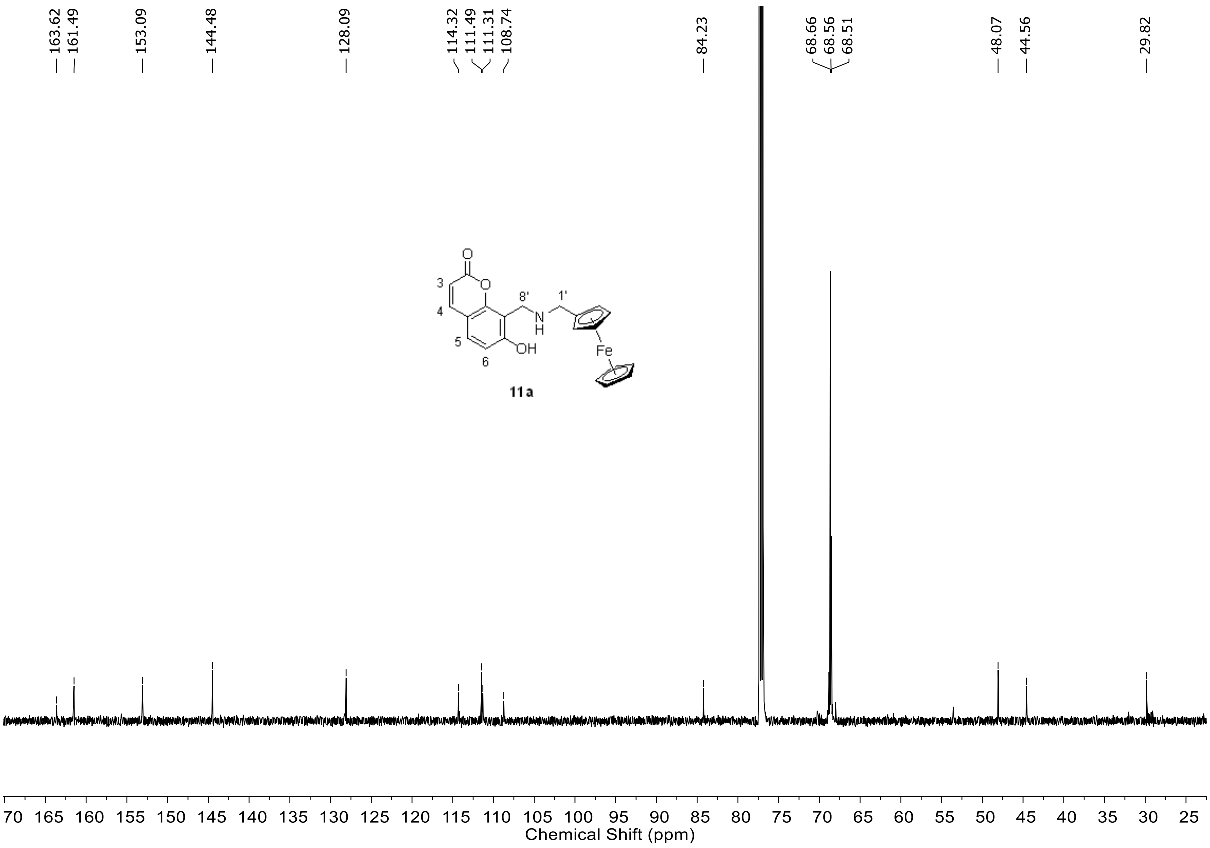

^{1}H NMR spectrum of compound **11b**

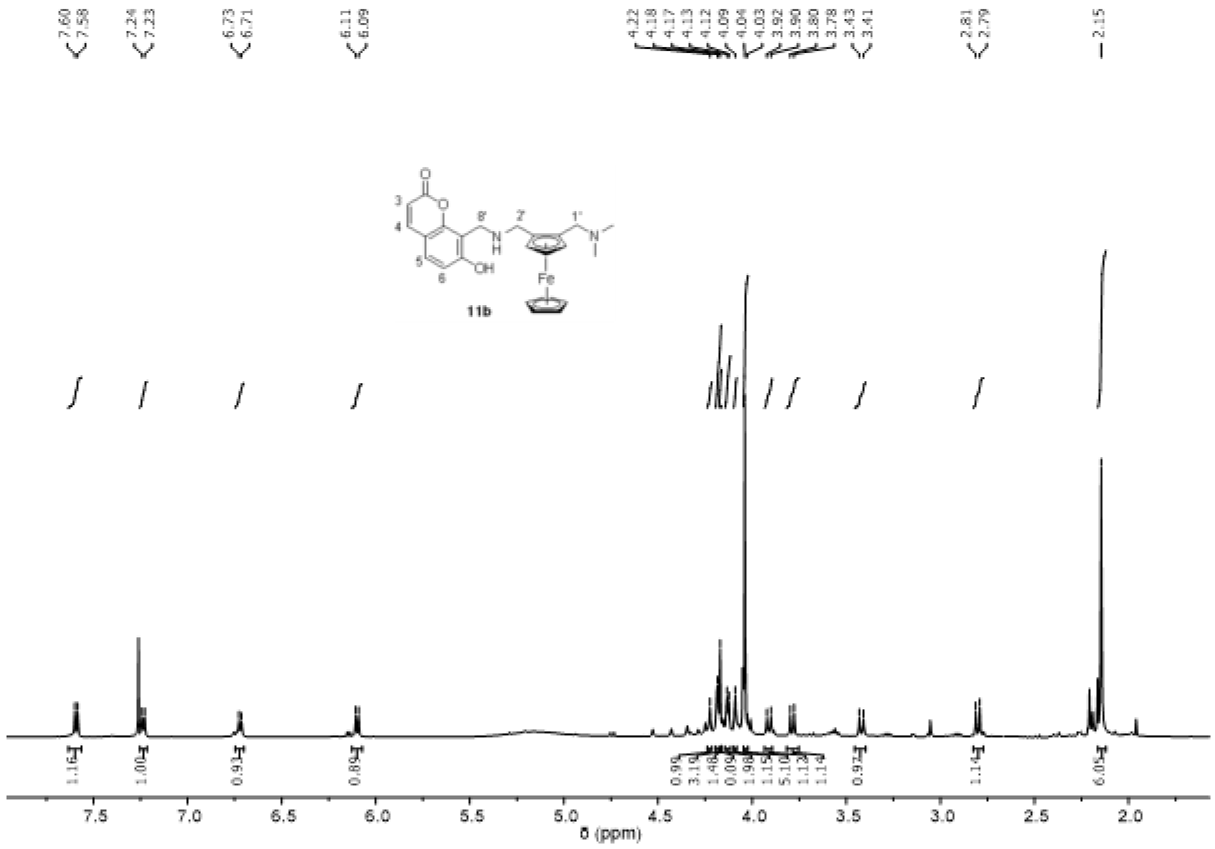

^{13}C NMR spectrum of compound **11b**

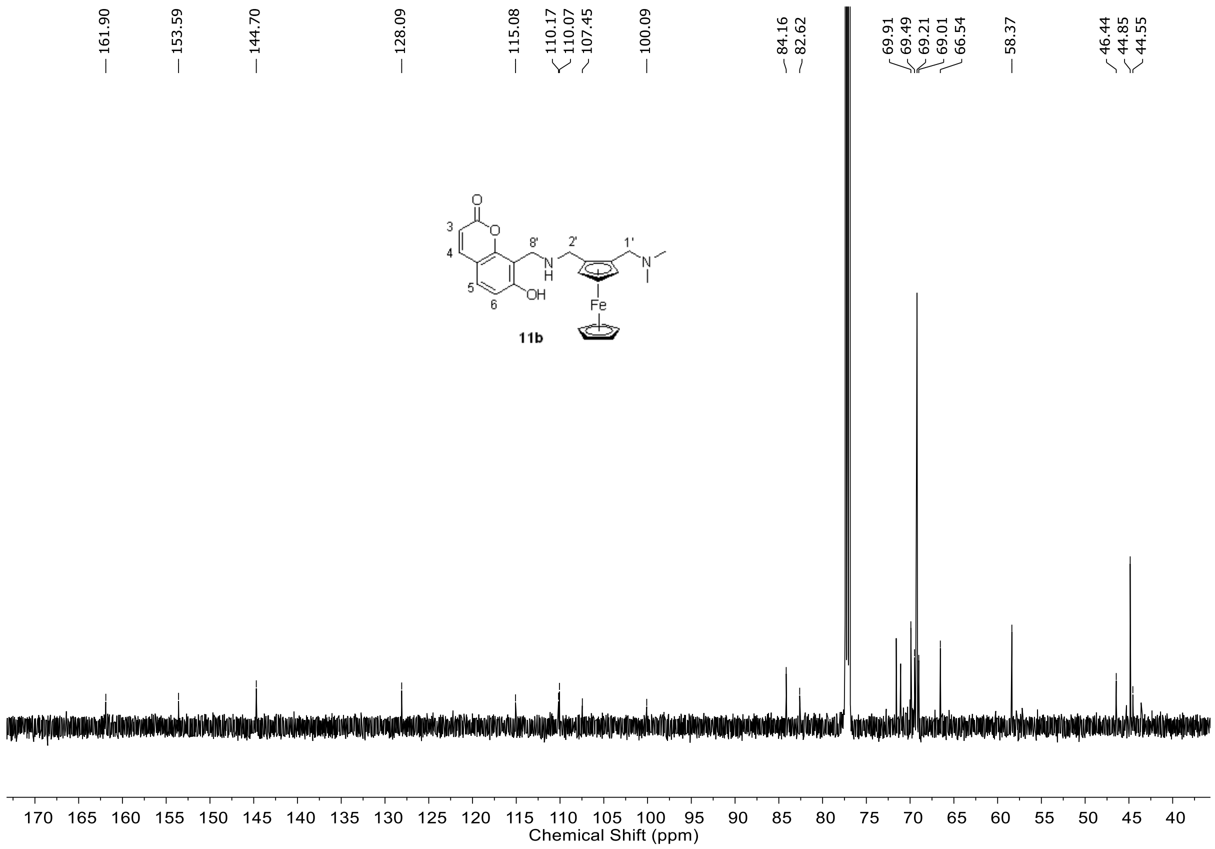

^{1}H NMR spectrum of compound **12a**

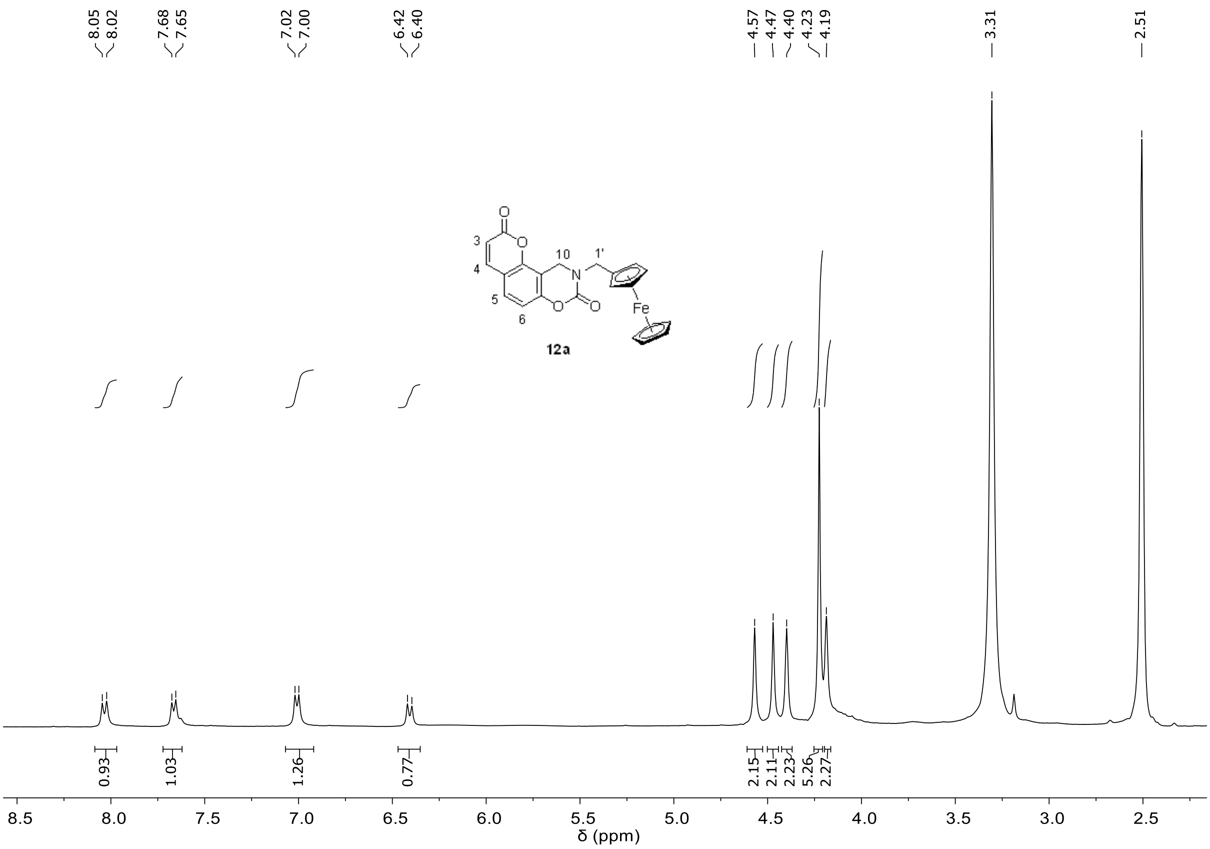

^{13}C NMR spectrum of compound **12a**

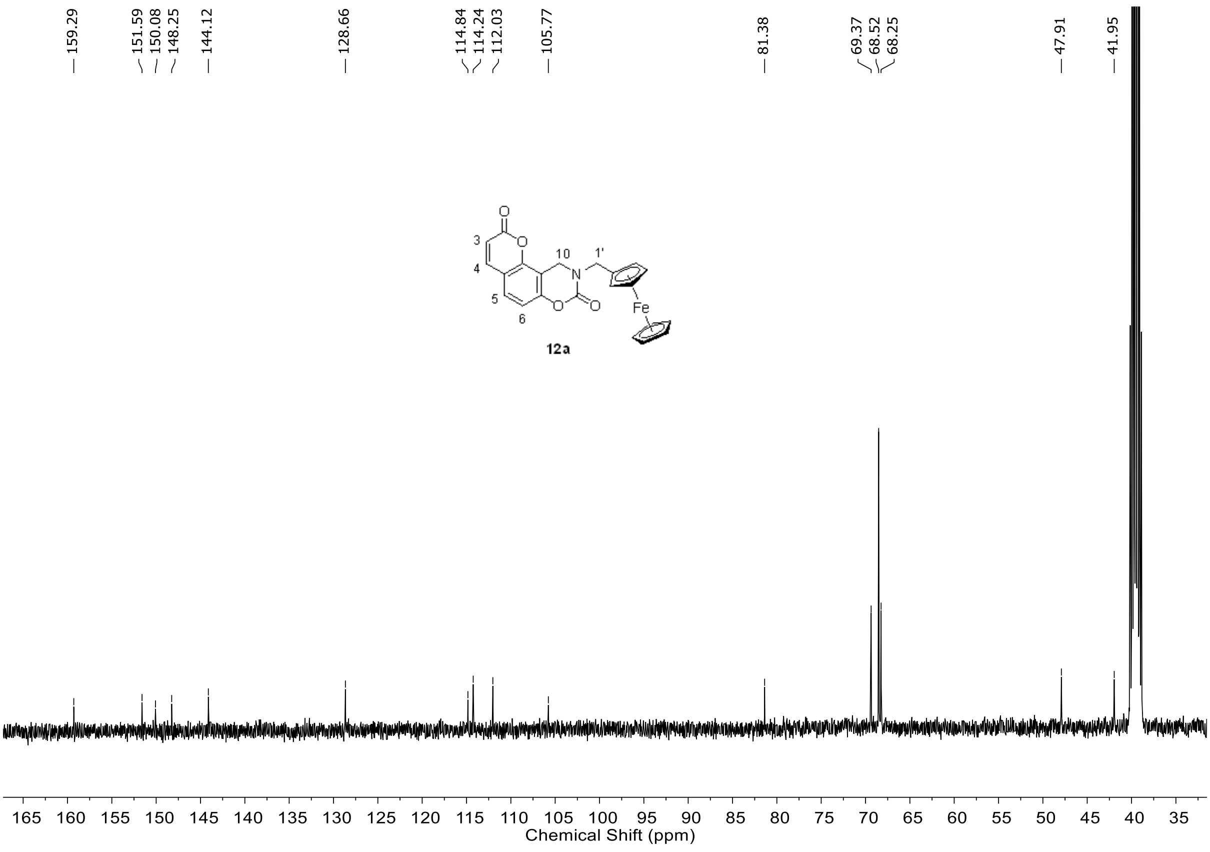

¹H NMR spectrum of compound **12b**

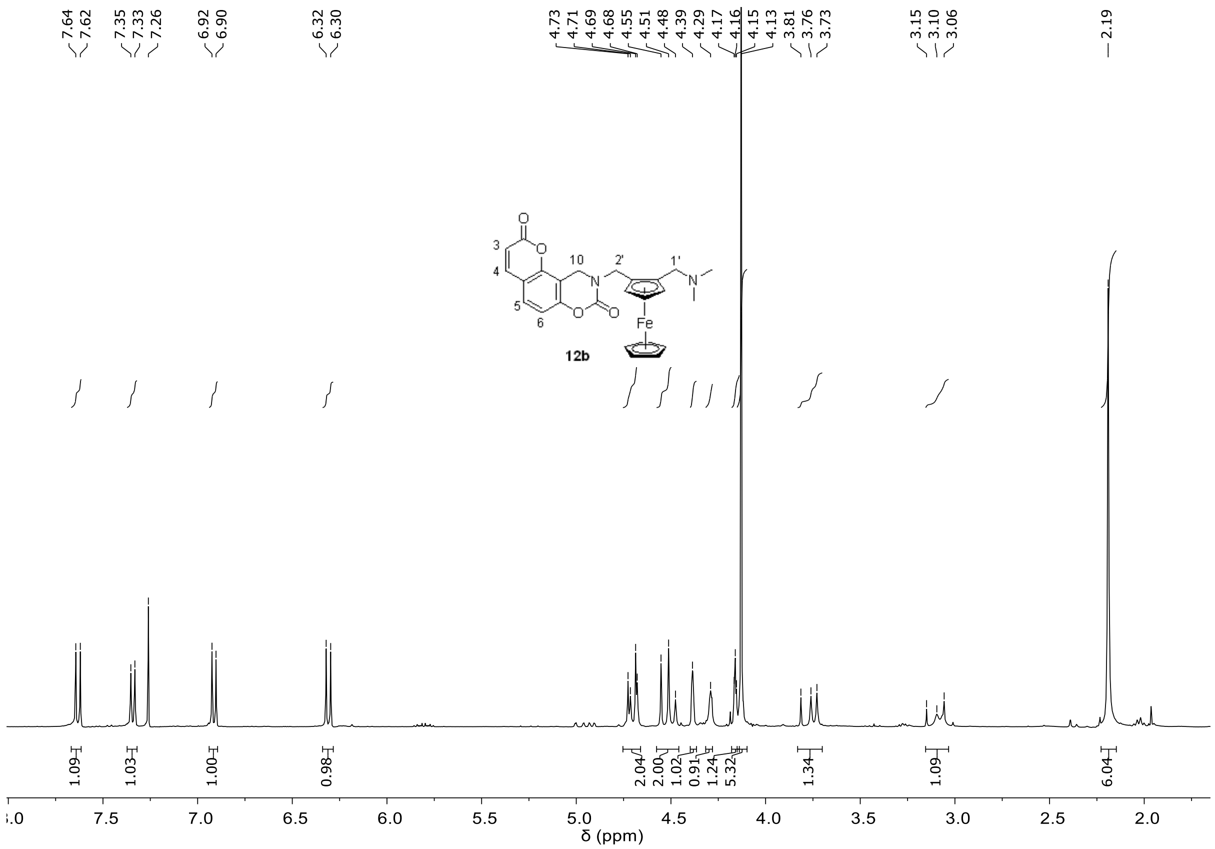

¹³C NMR spectrum of compound **12b**

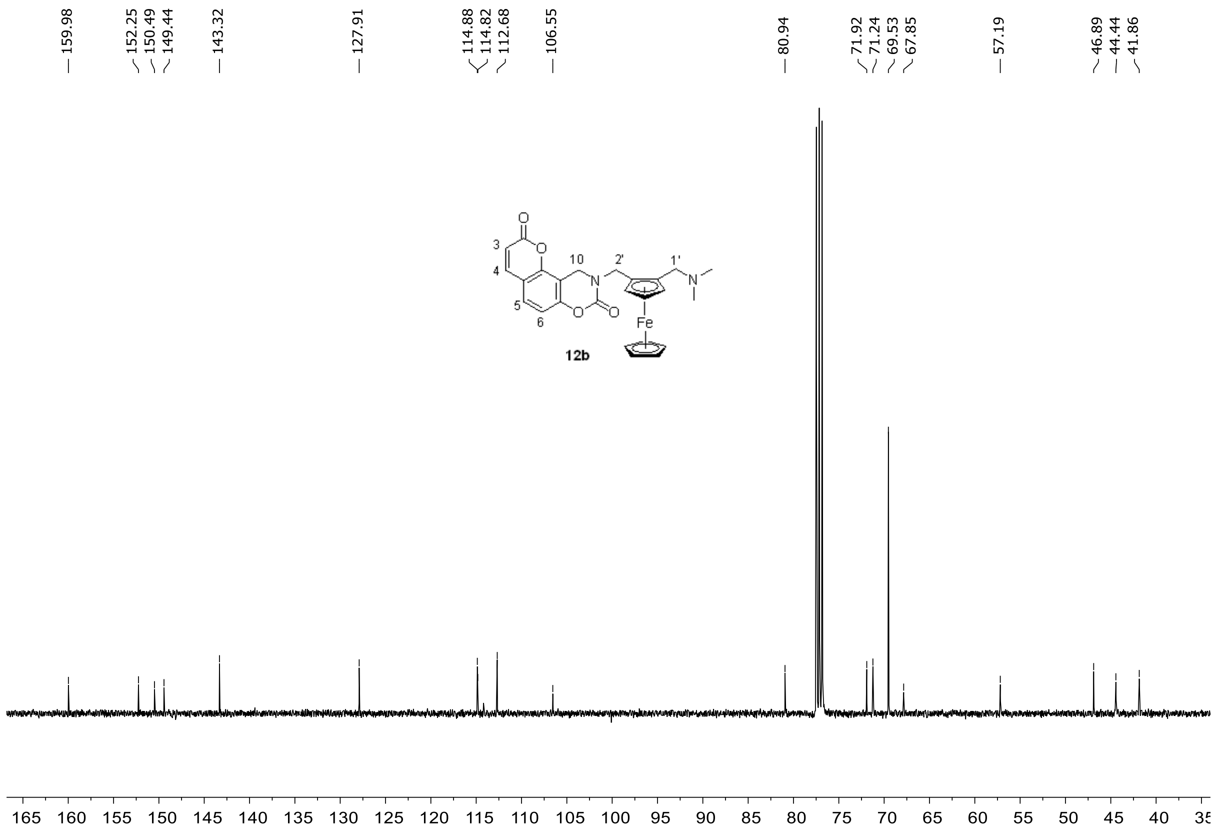

^{1}H NMR spectrum of compound **13**

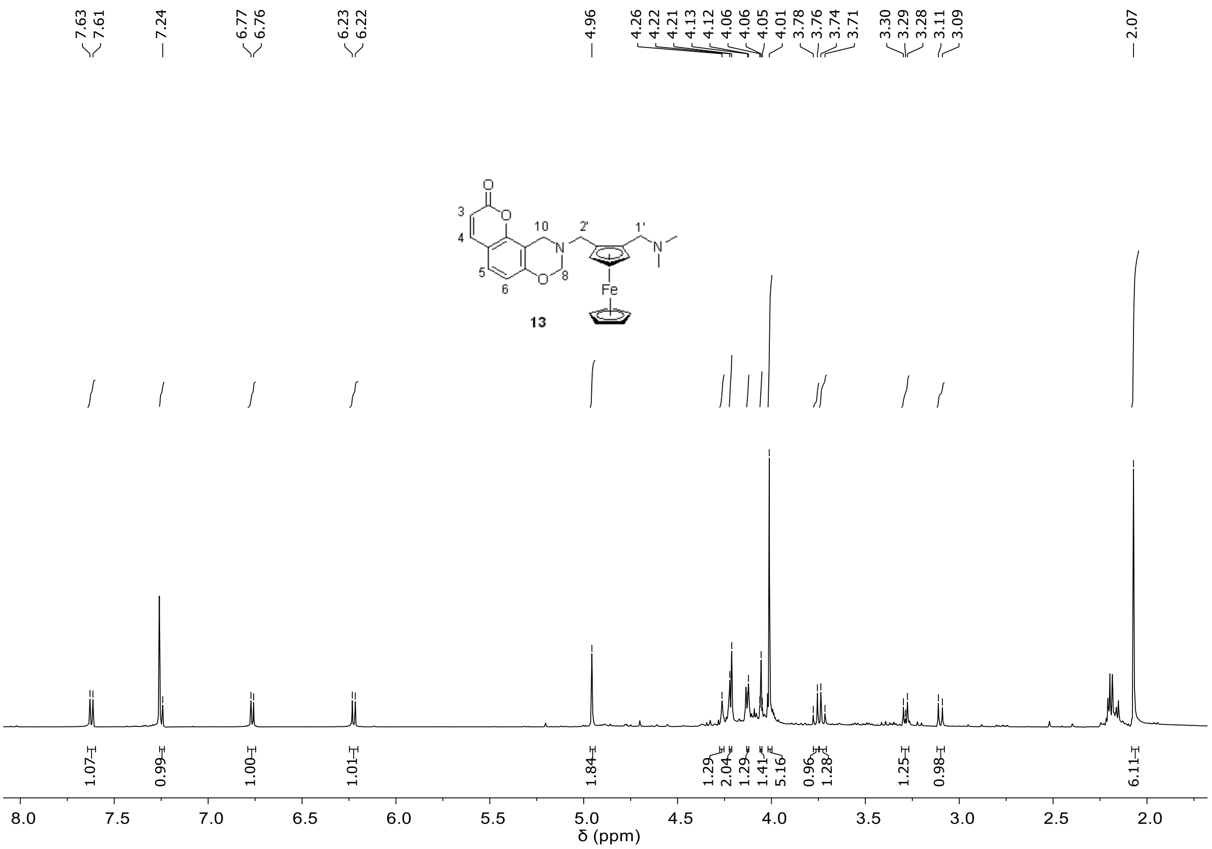

^{13}C NMR spectrum of compound **13**

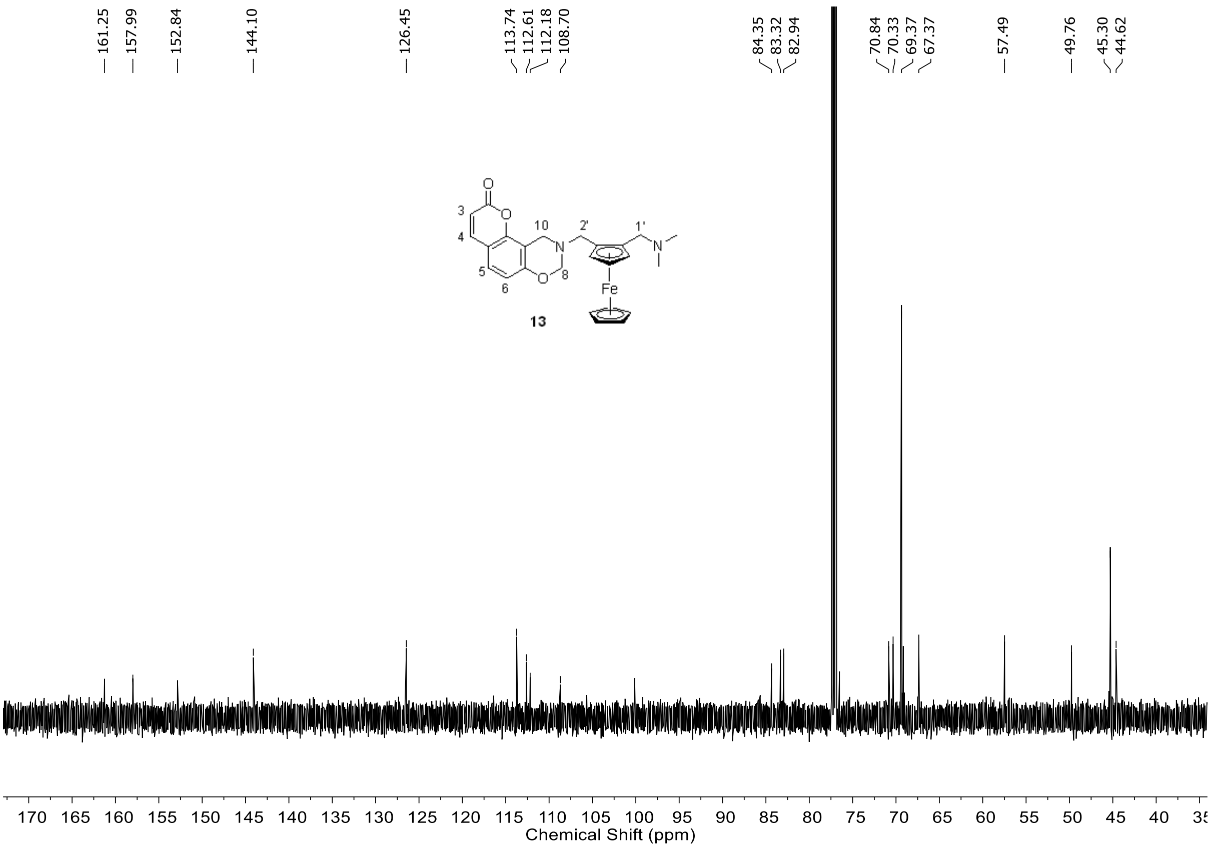

^{1}H NMR spectrum of compound **15a**

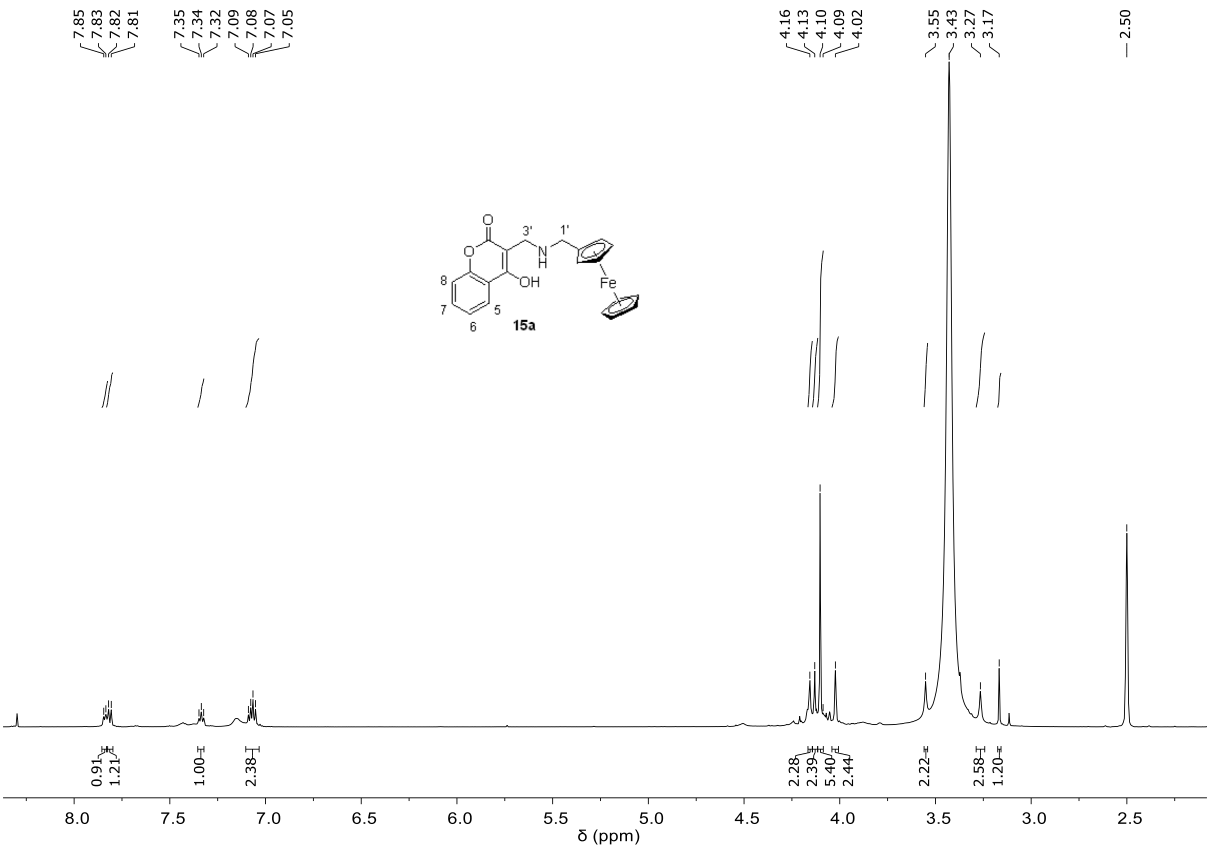

^{13}C NMR spectrum of compound **15a**

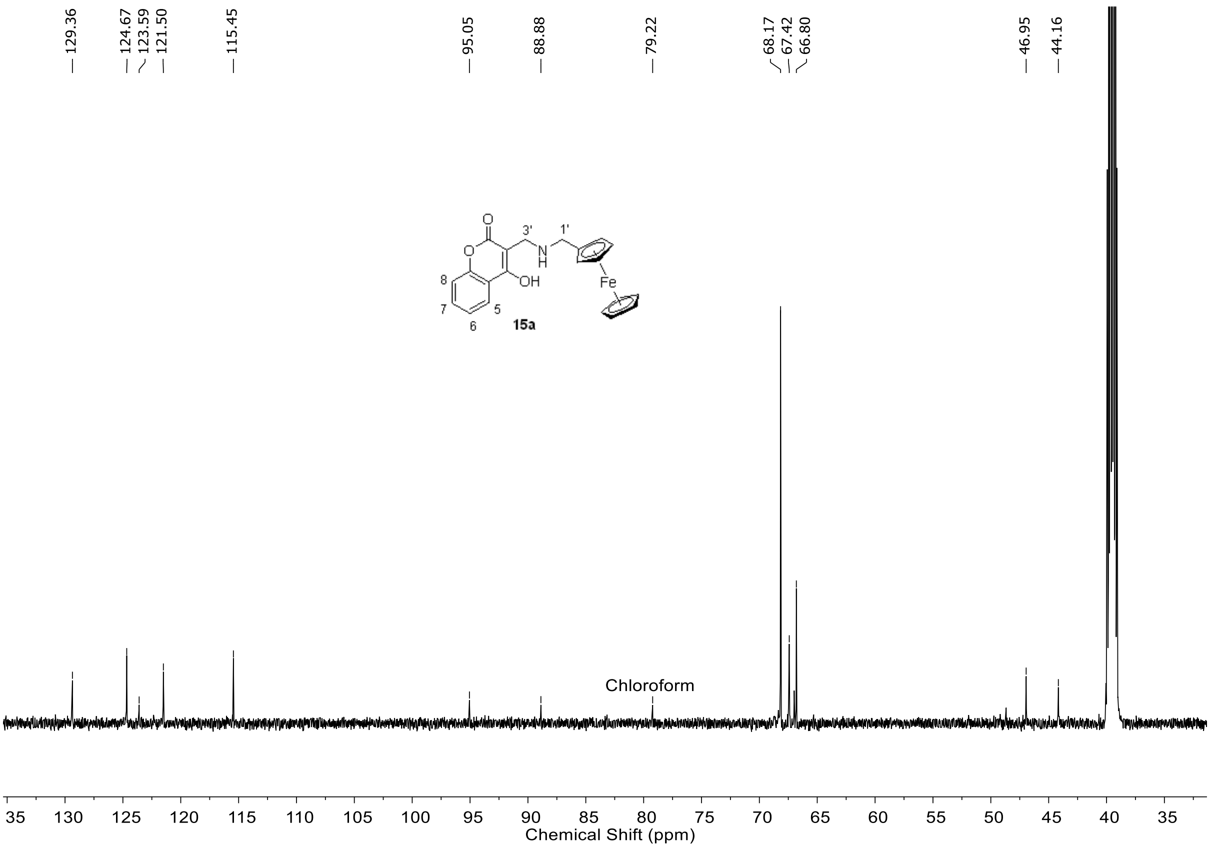

^{1}H NMR spectrum of compound **15b**

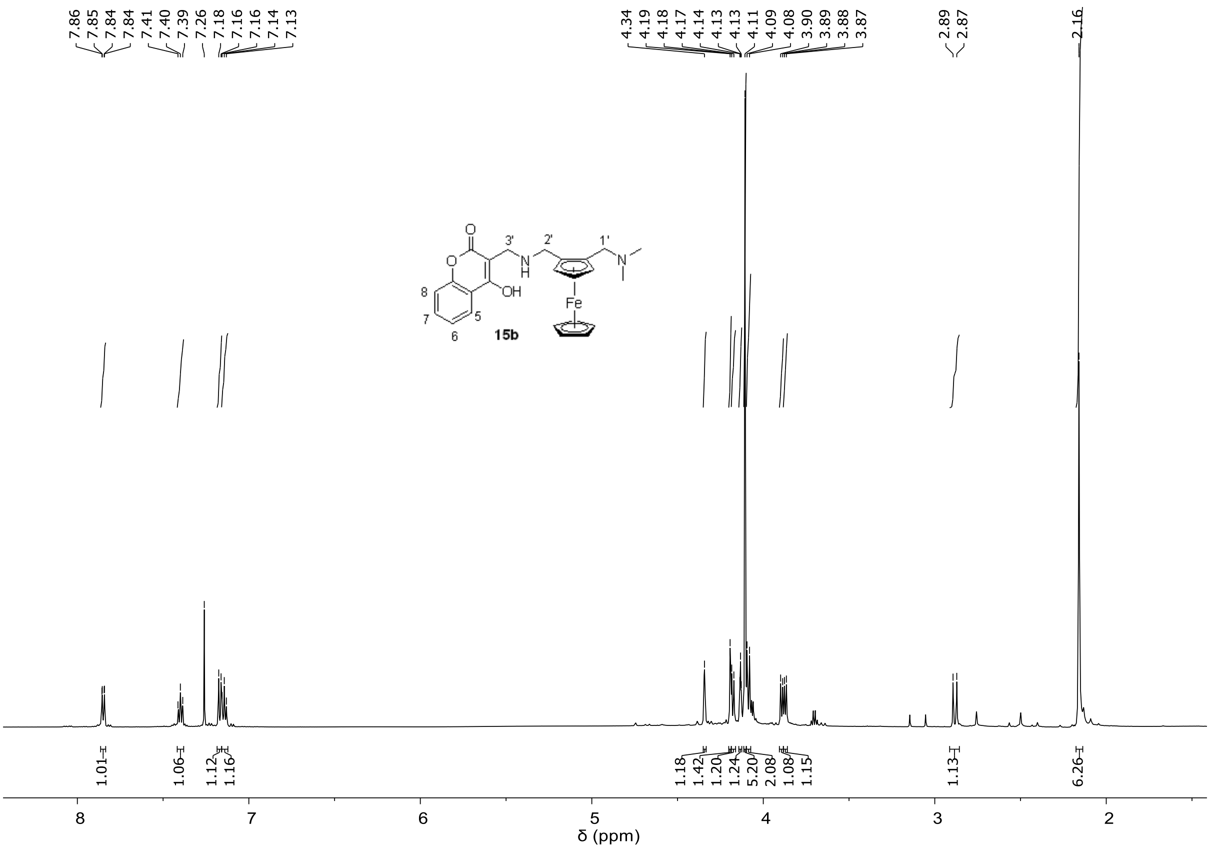

^{13}C NMR spectrum of compound **15b**

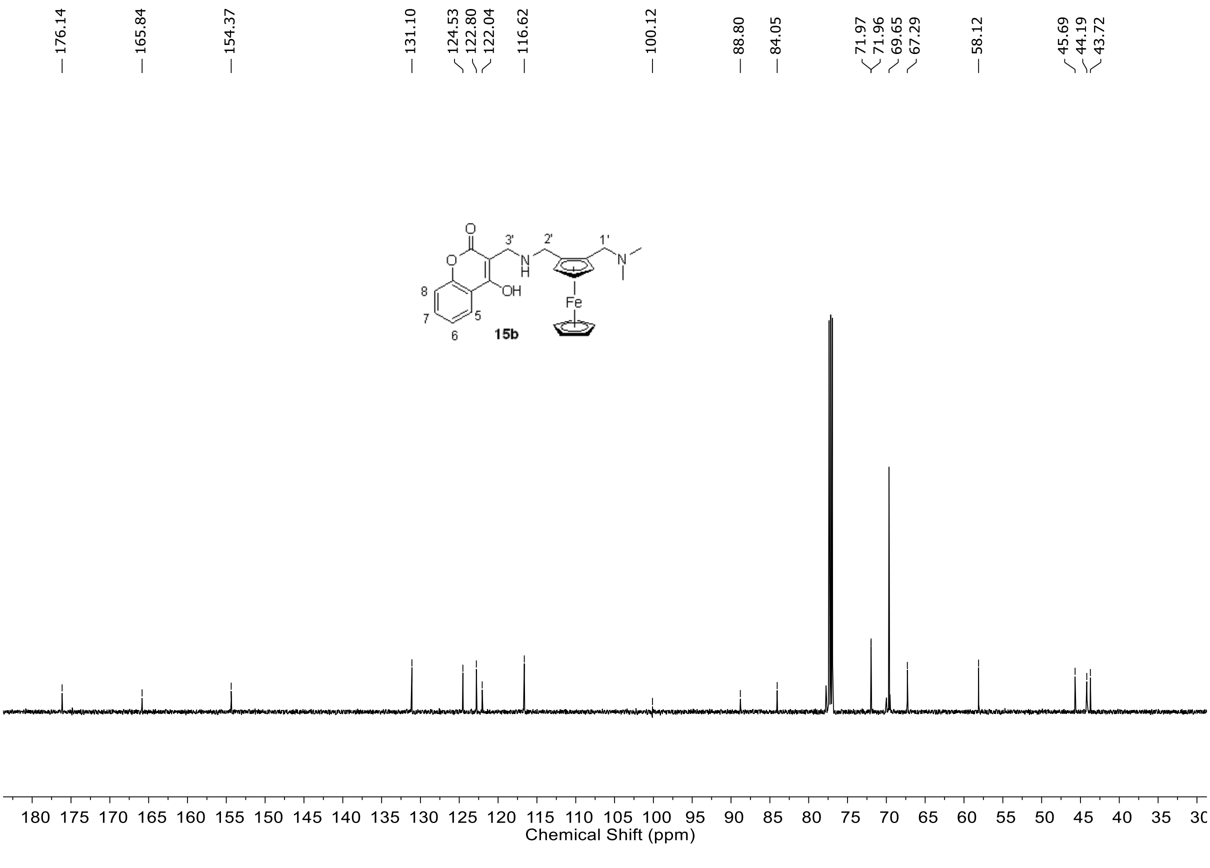

^{1}H NMR spectrum of compound **15c**

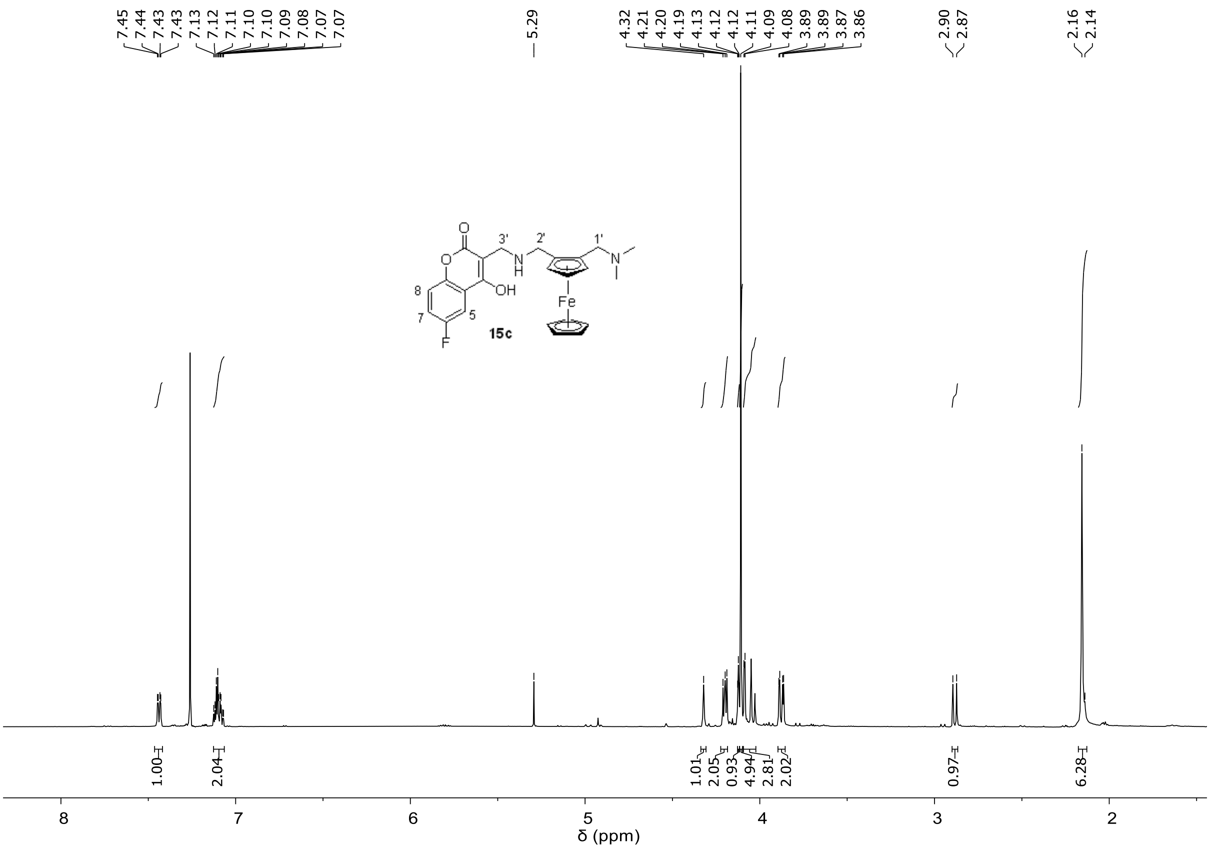

^{13}C NMR spectrum of compound **15c**

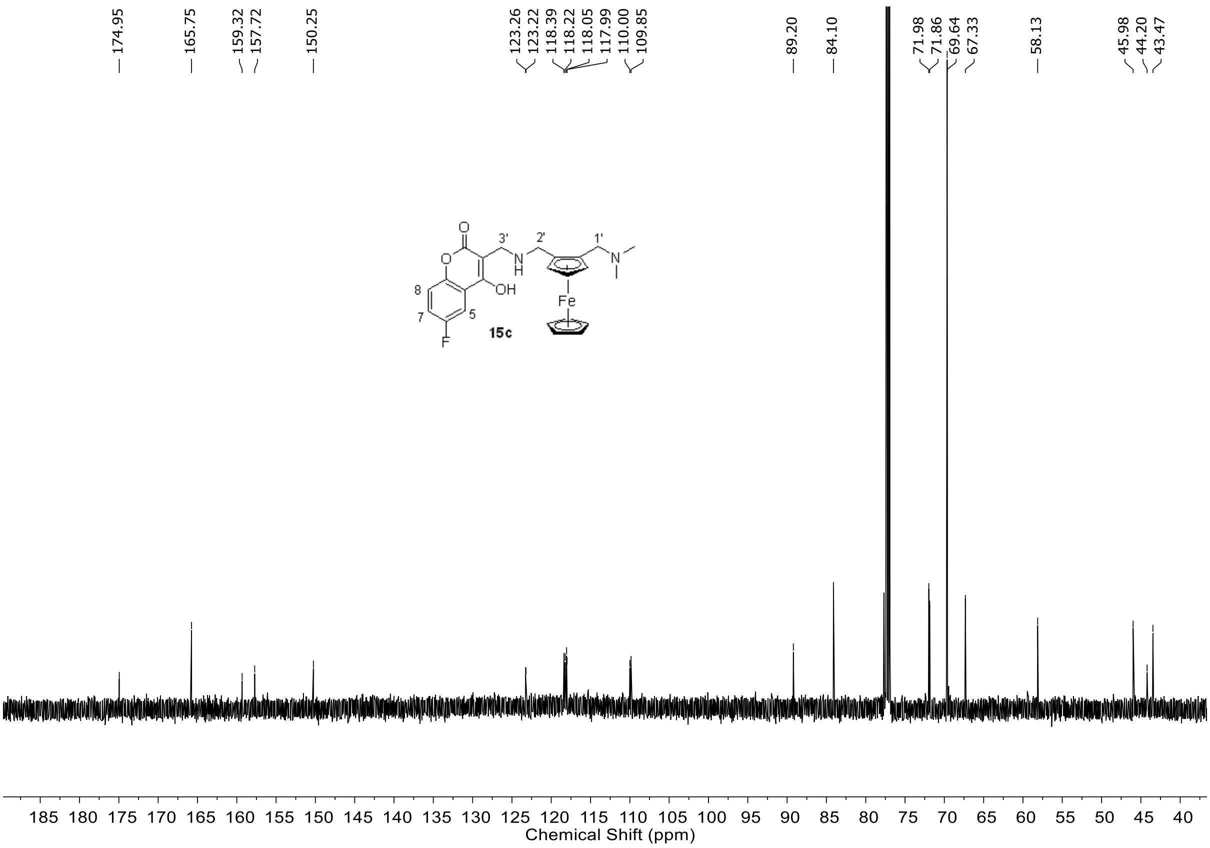

^{1}H NMR spectrum of compound **15d**

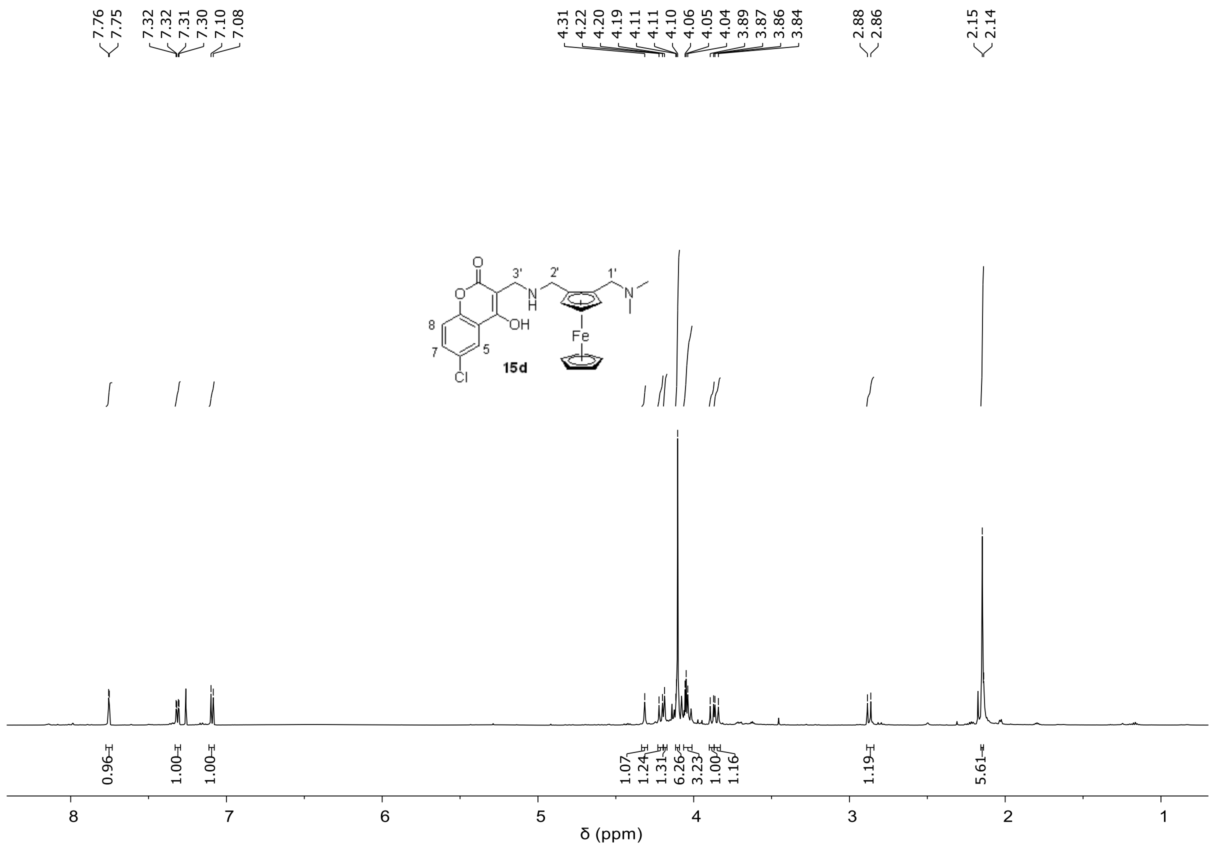

^{13}C NMR spectrum of compound **15d**

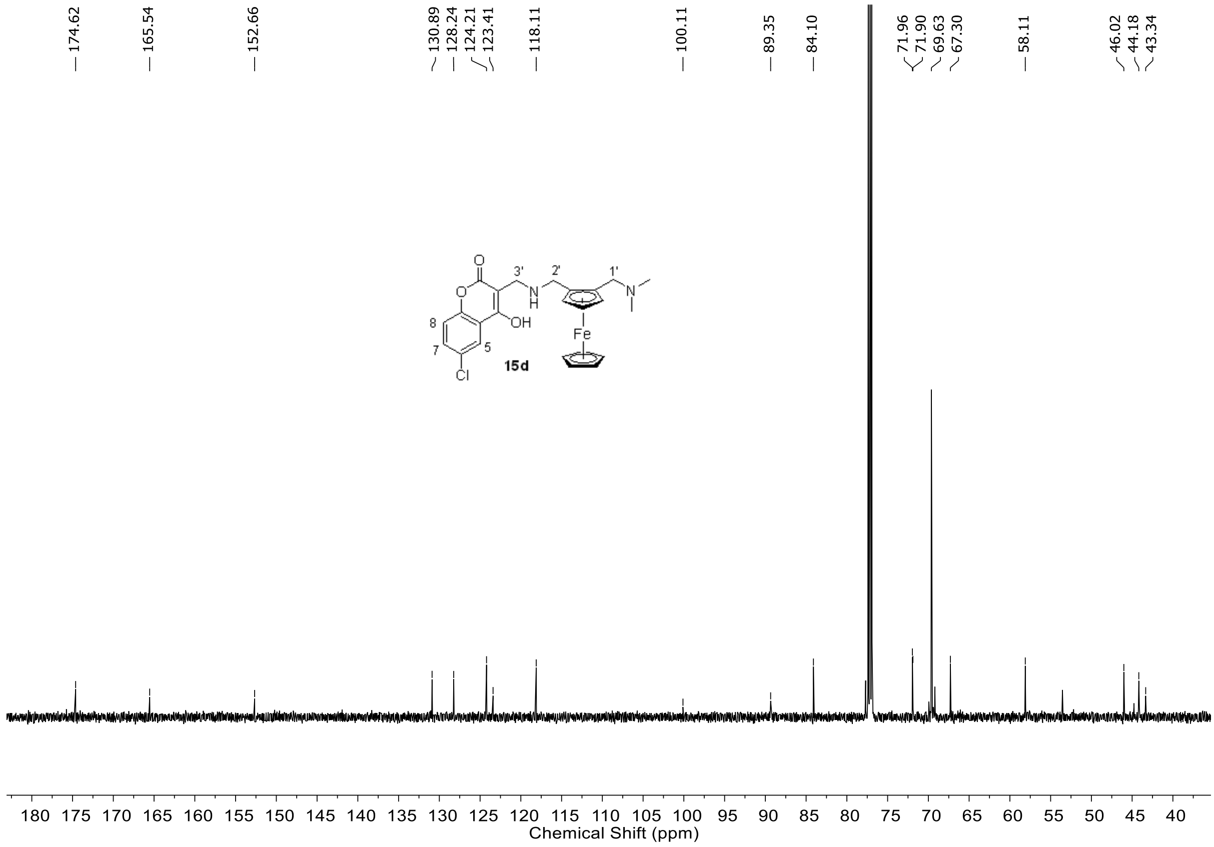

^1H NMR spectrum of compound **16a**

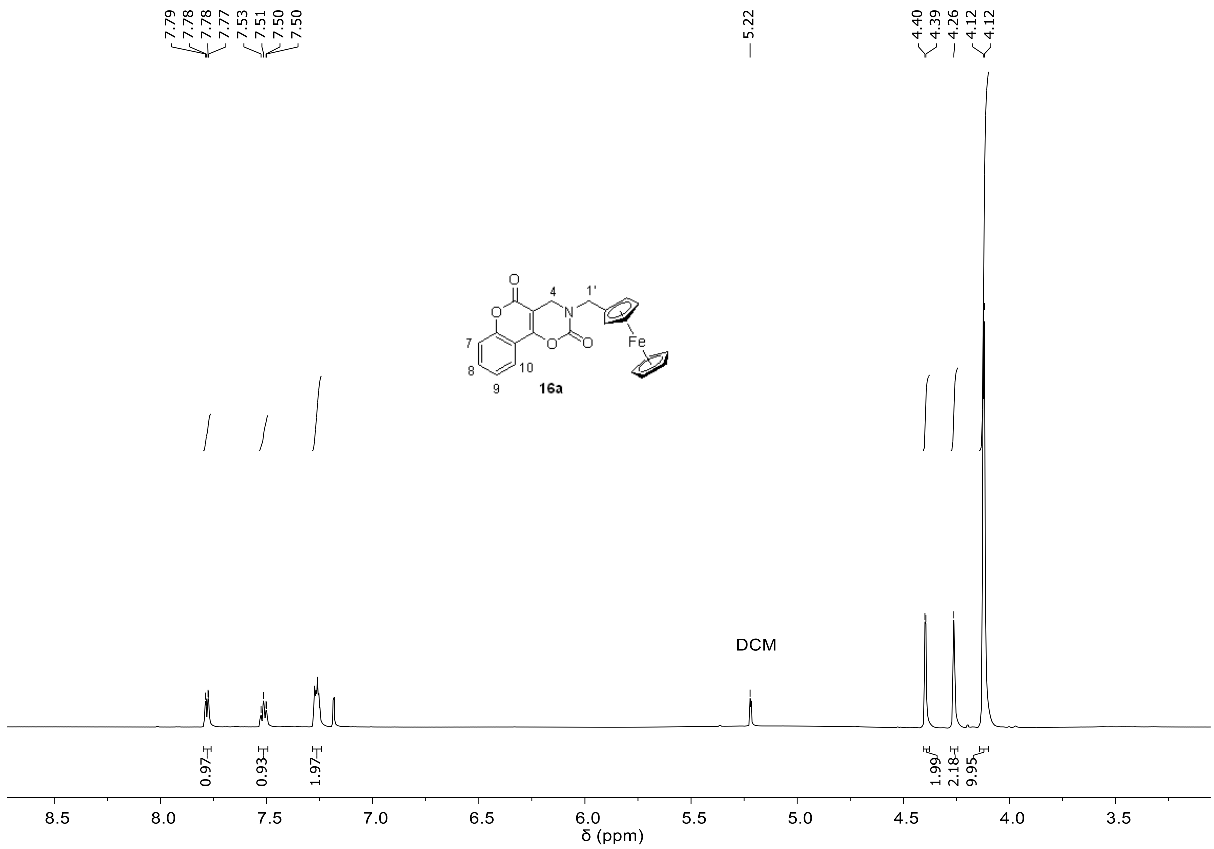

^{13}C NMR spectrum of compound **16a**

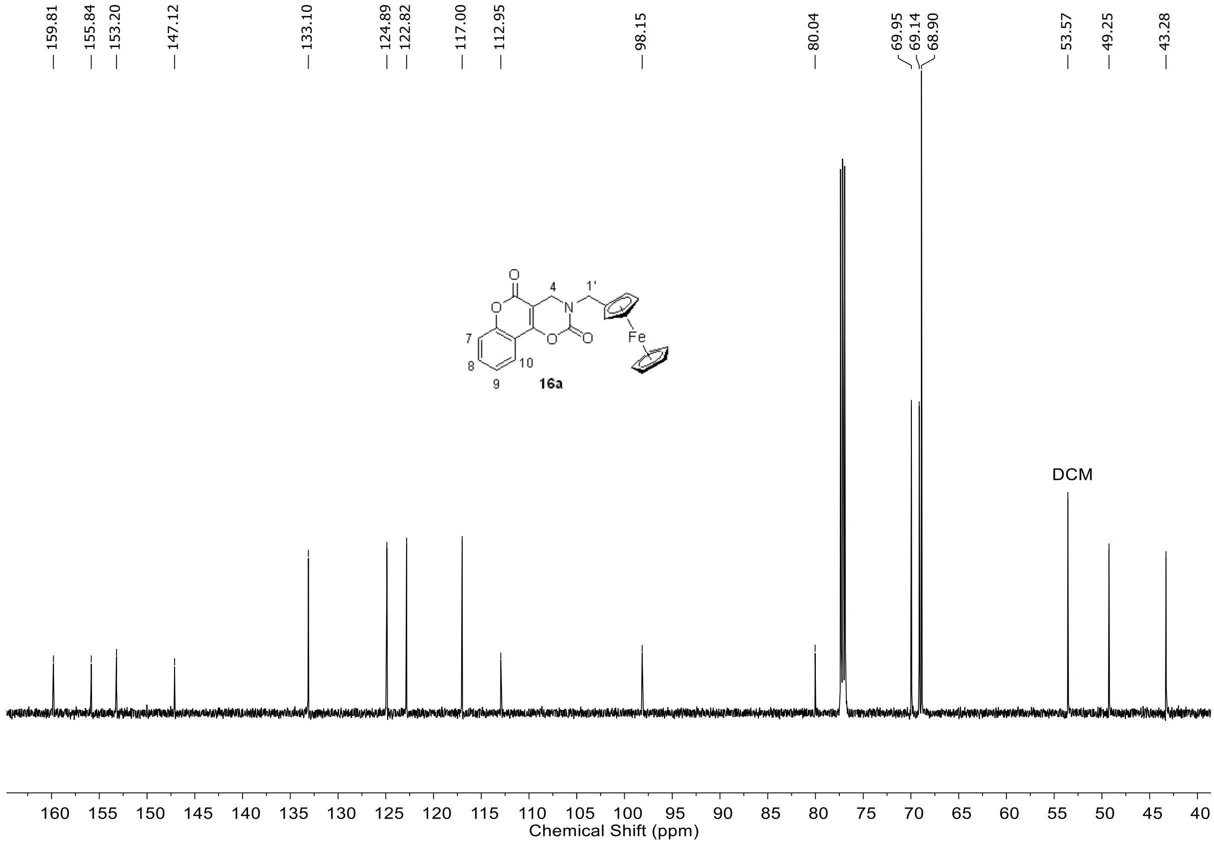

^{1}H NMR spectrum of compound **16b**

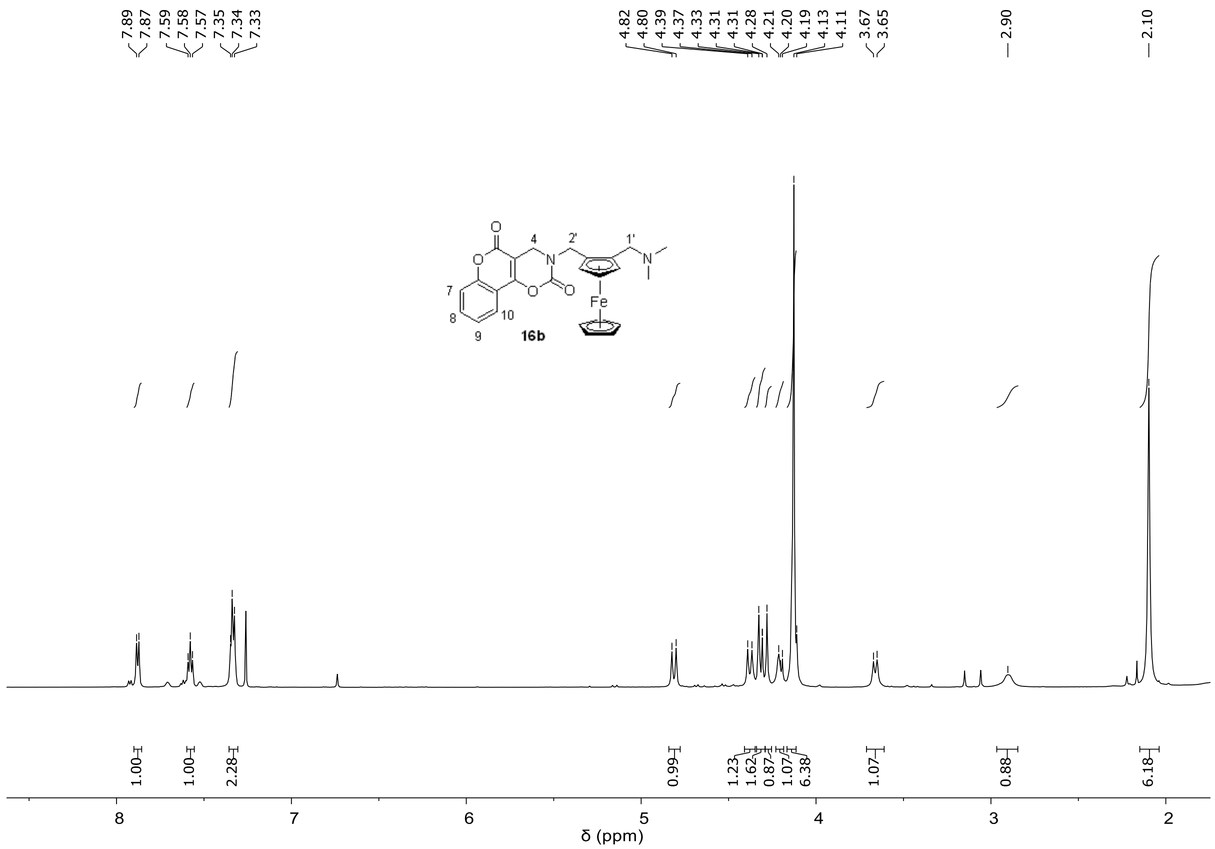

^{13}C NMR spectrum of compound **16b**

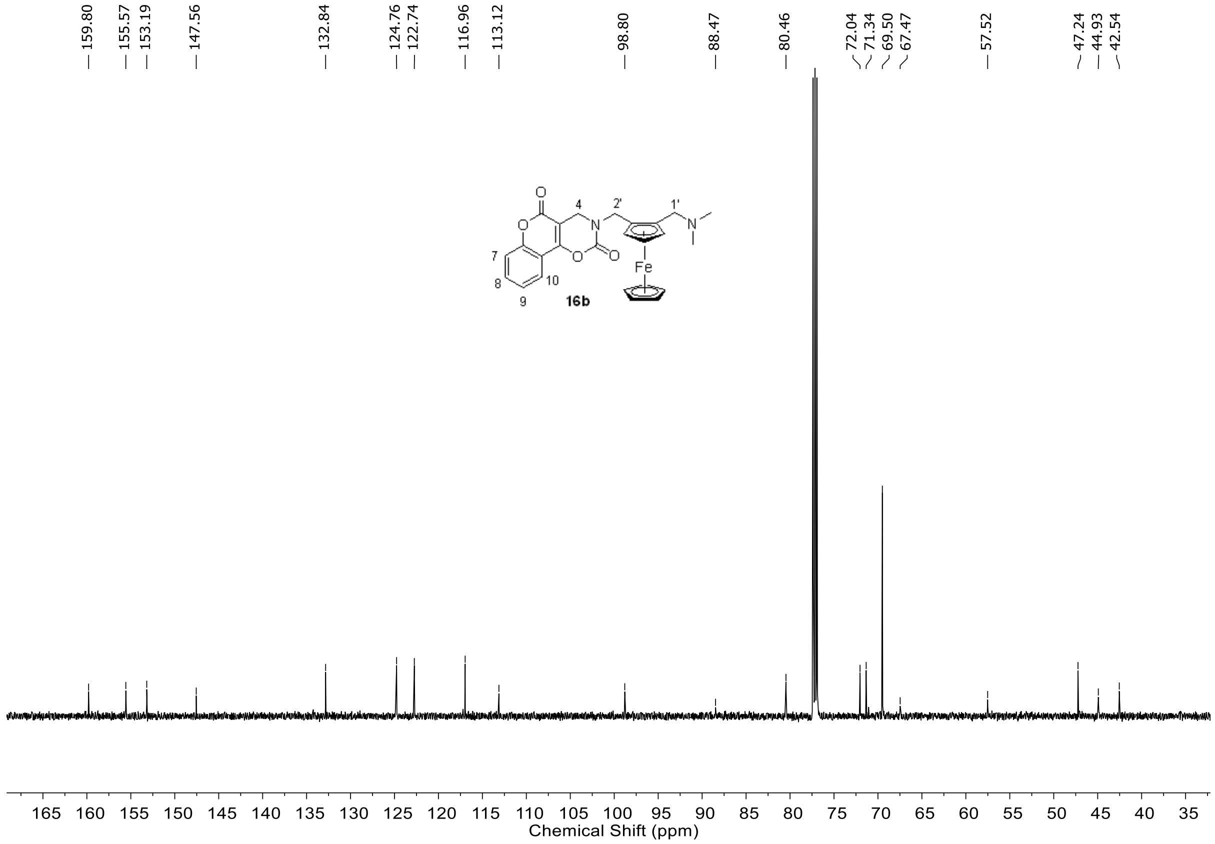

^{1}H NMR spectrum of compound **16c**

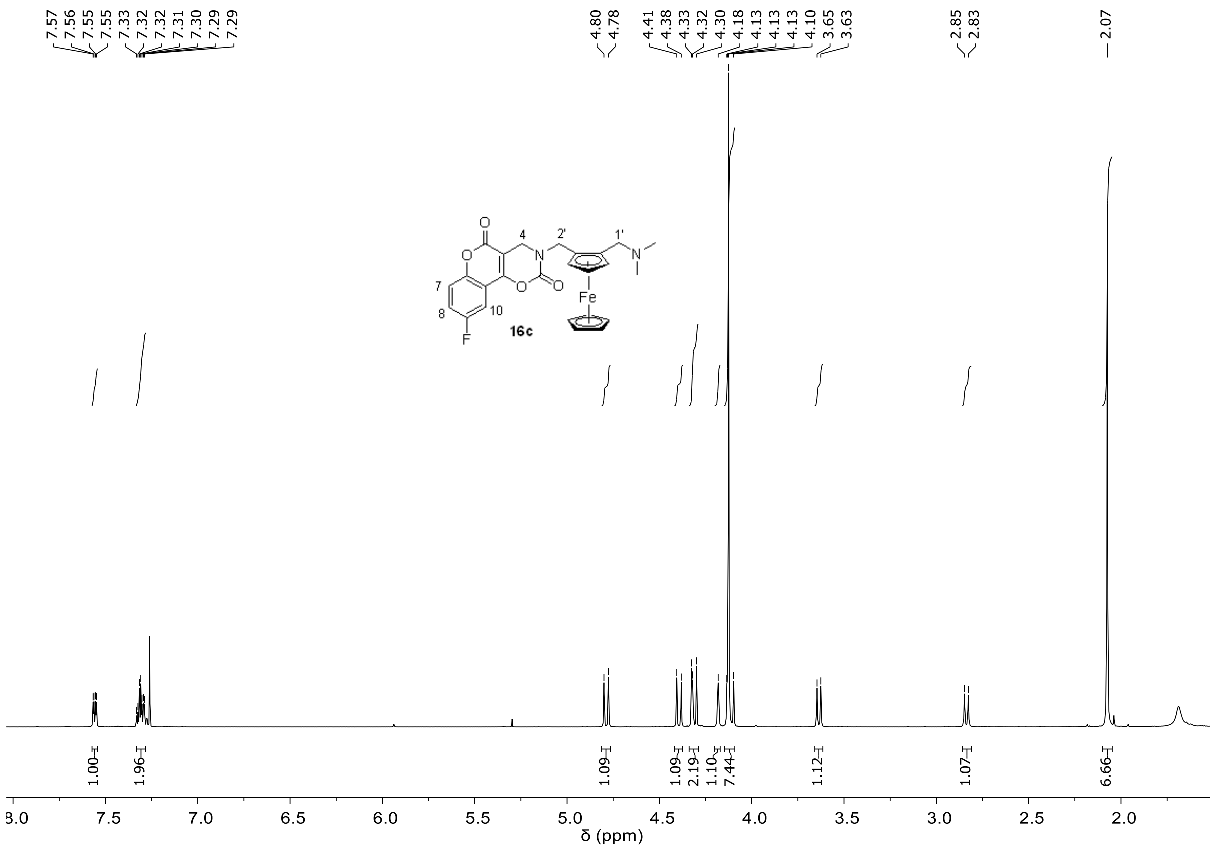

^{13}C NMR spectrum of compound **16c**

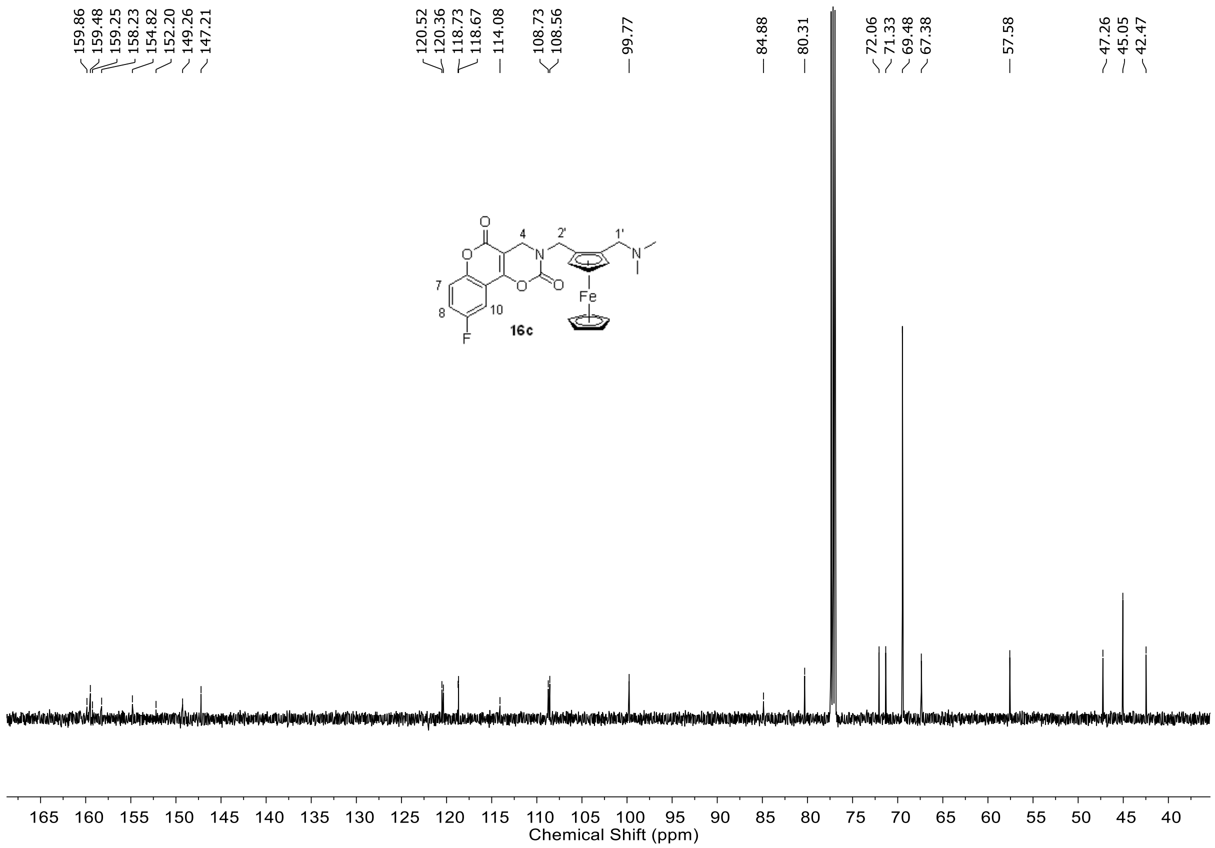

^{1}H NMR spectrum of compound **16d**

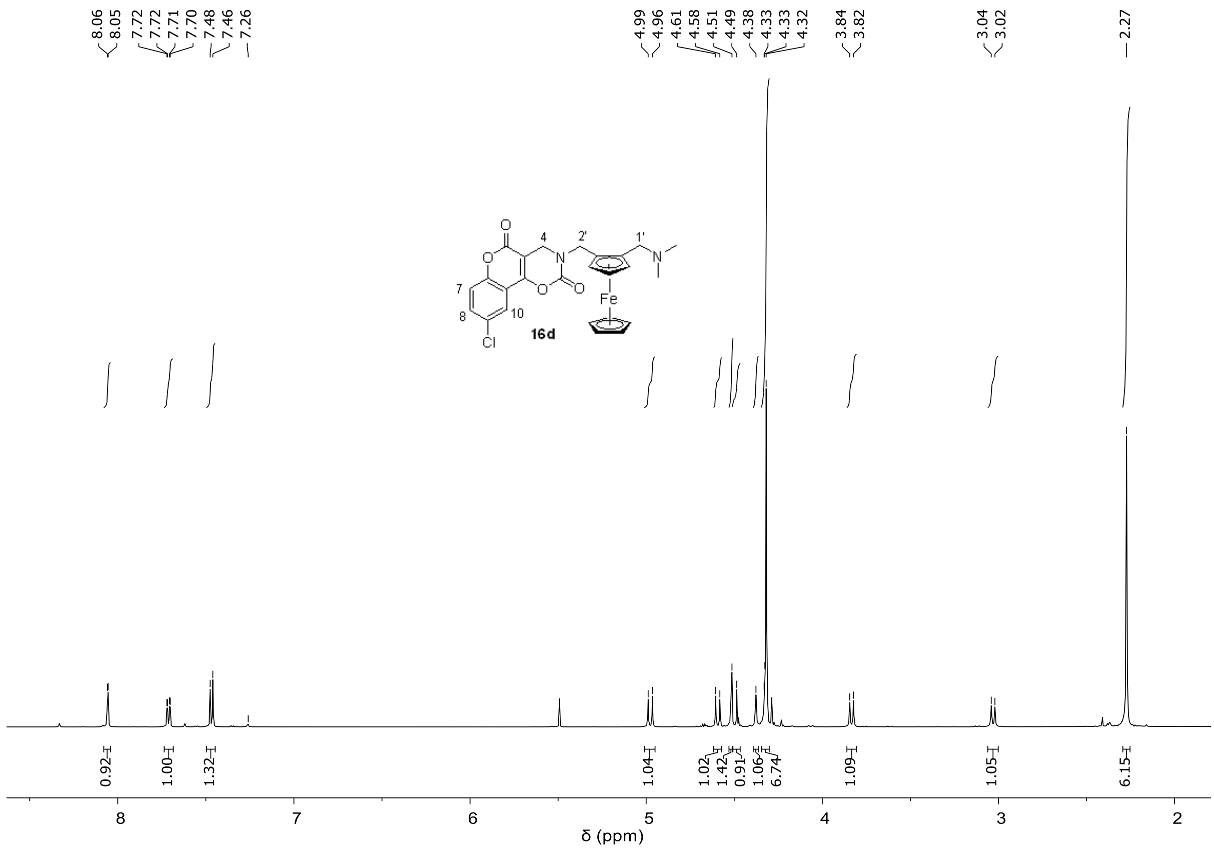

^{13}C NMR spectrum of compound **16d**

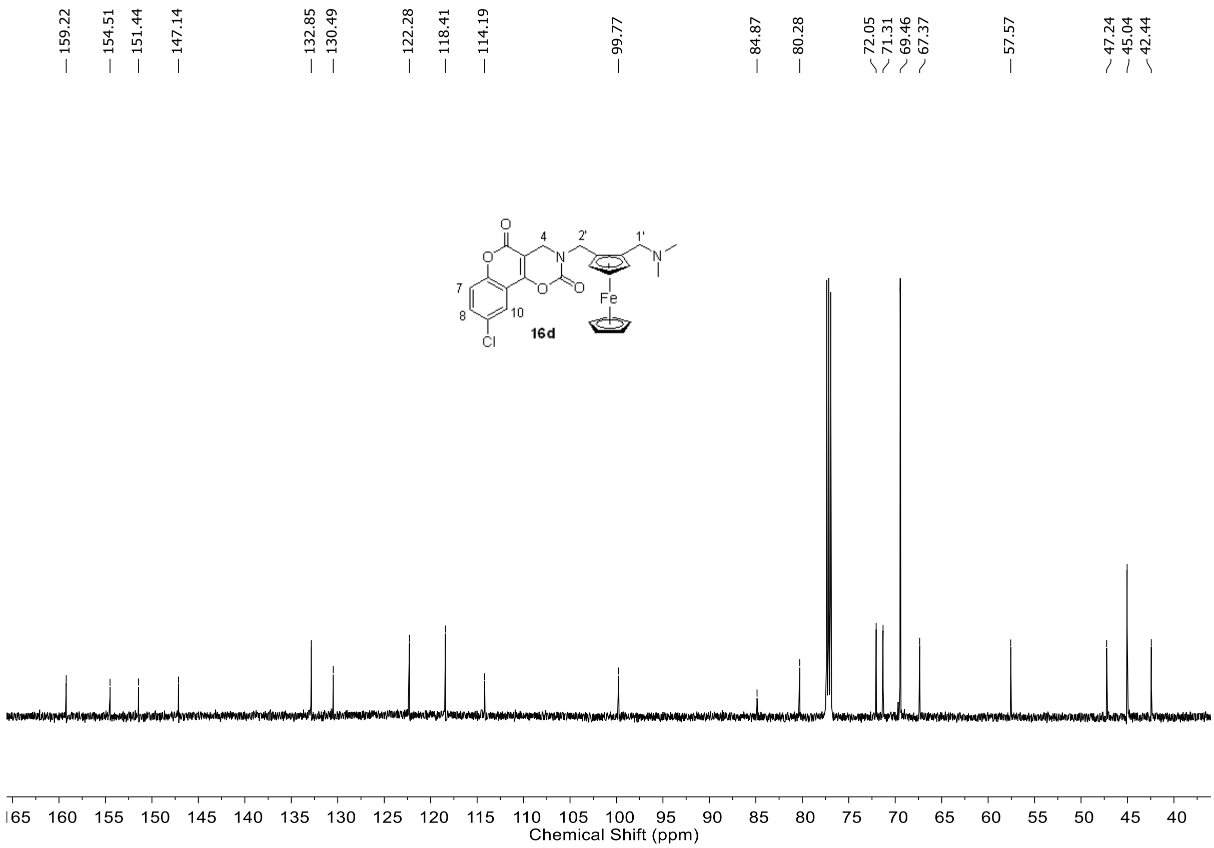

^{1}H NMR spectrum of compound **19**

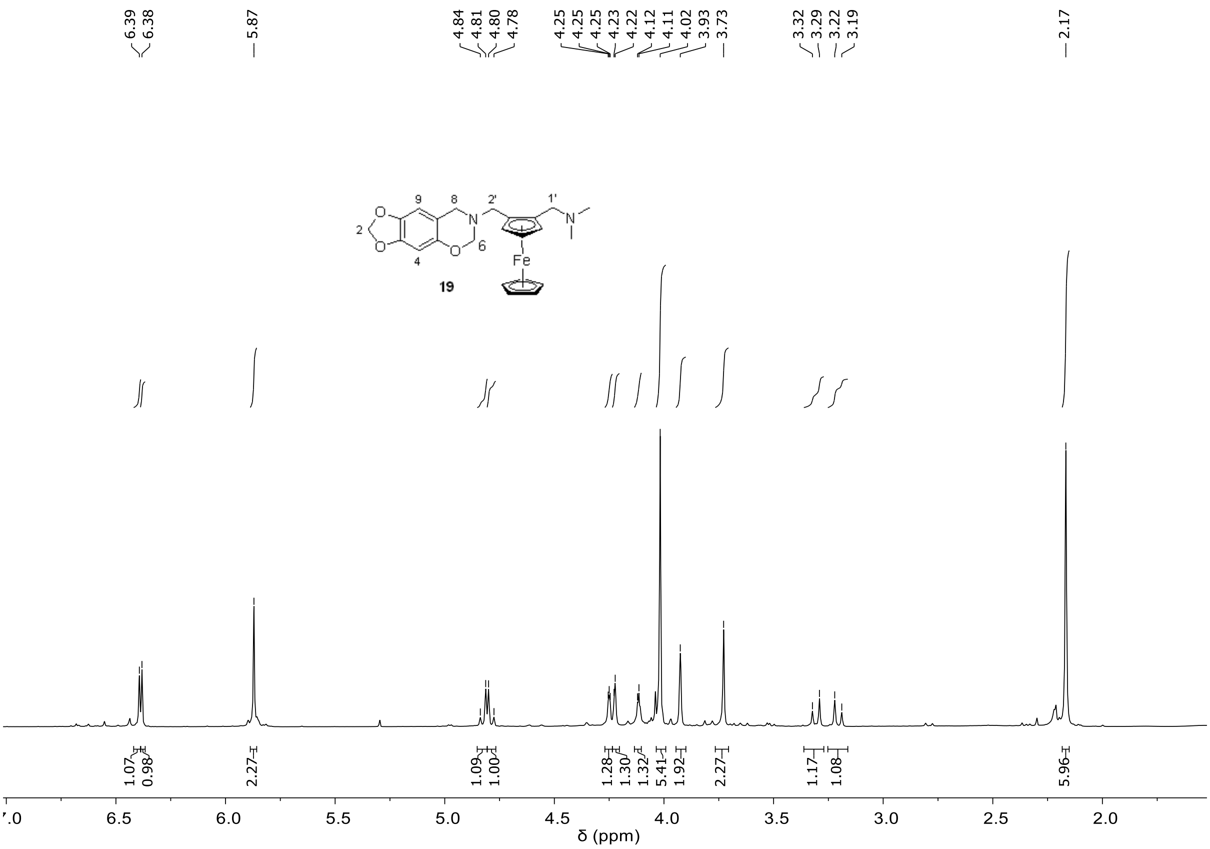

^{13}C NMR spectrum of compound **19**

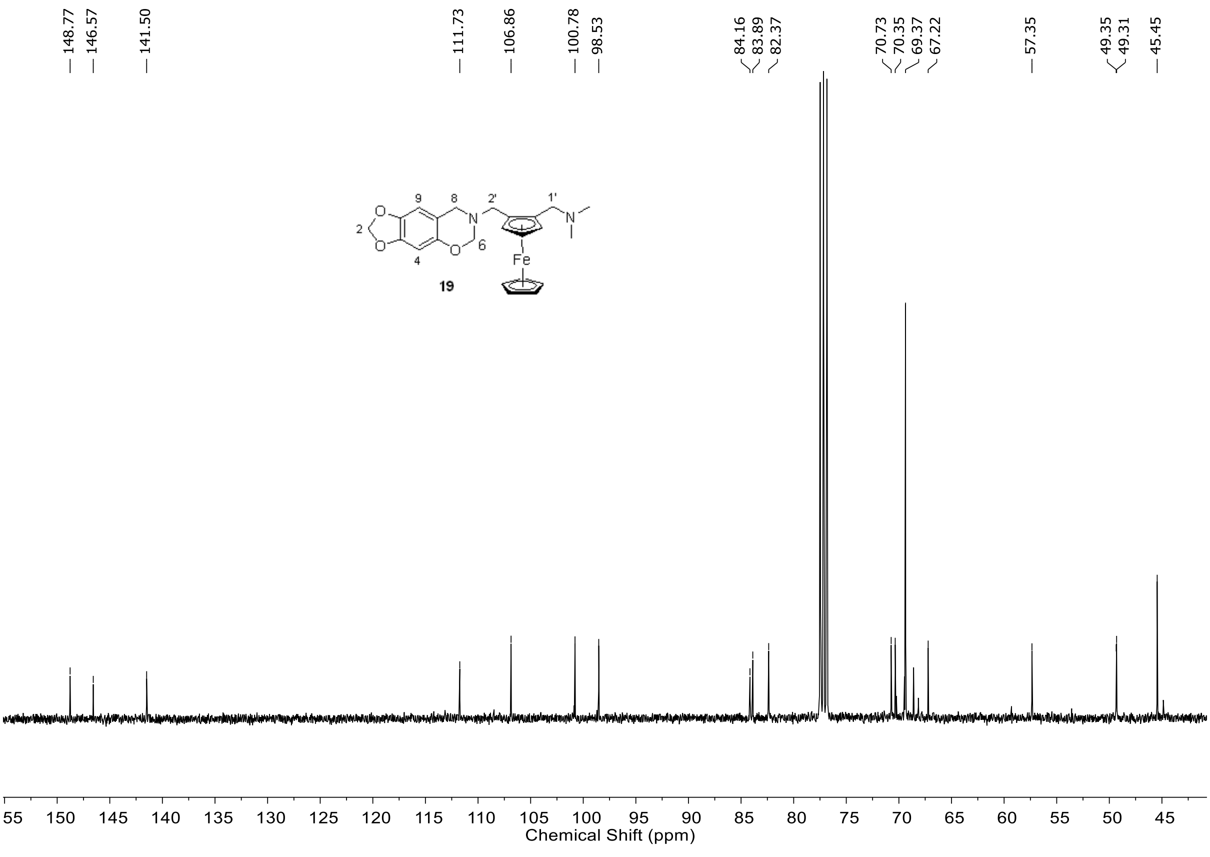

Representative COSY NMR spectrum of compound **12b**

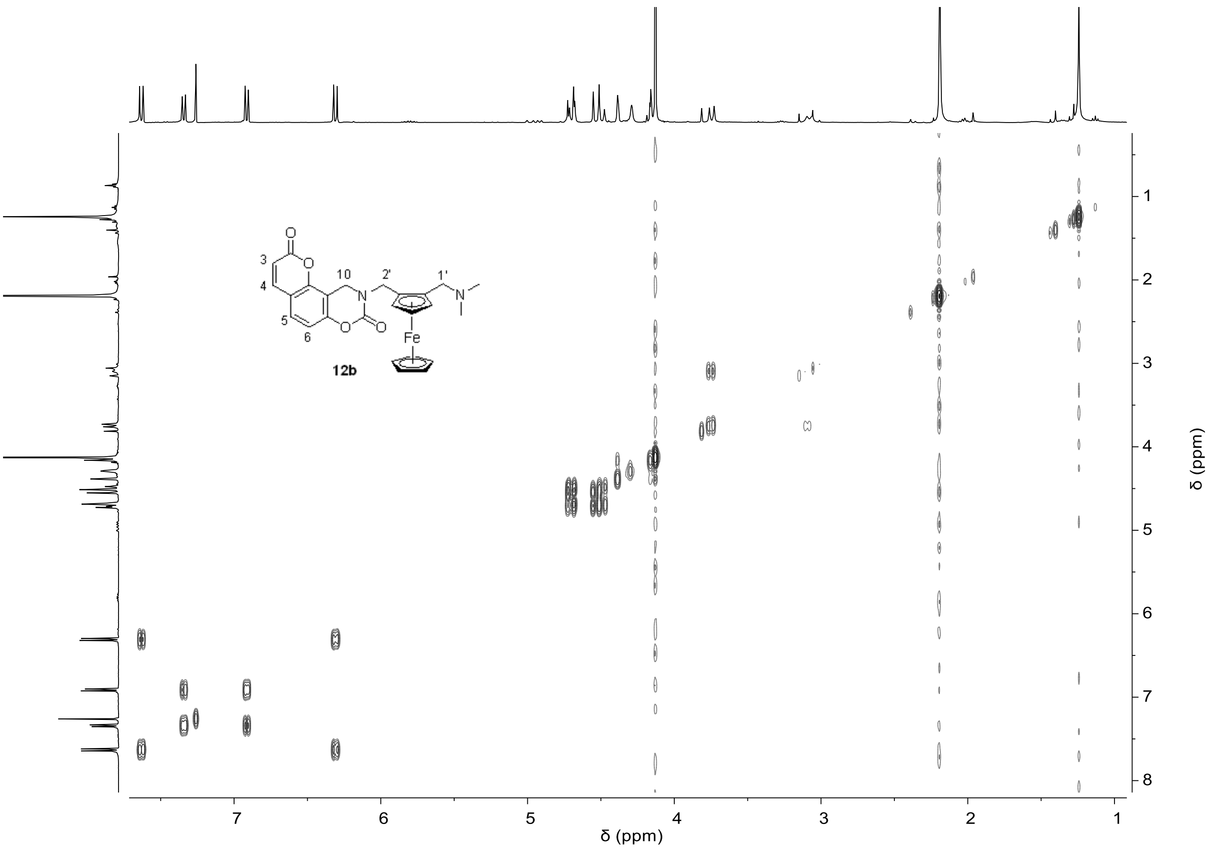

Representative HSQC spectrum of compound **12b**

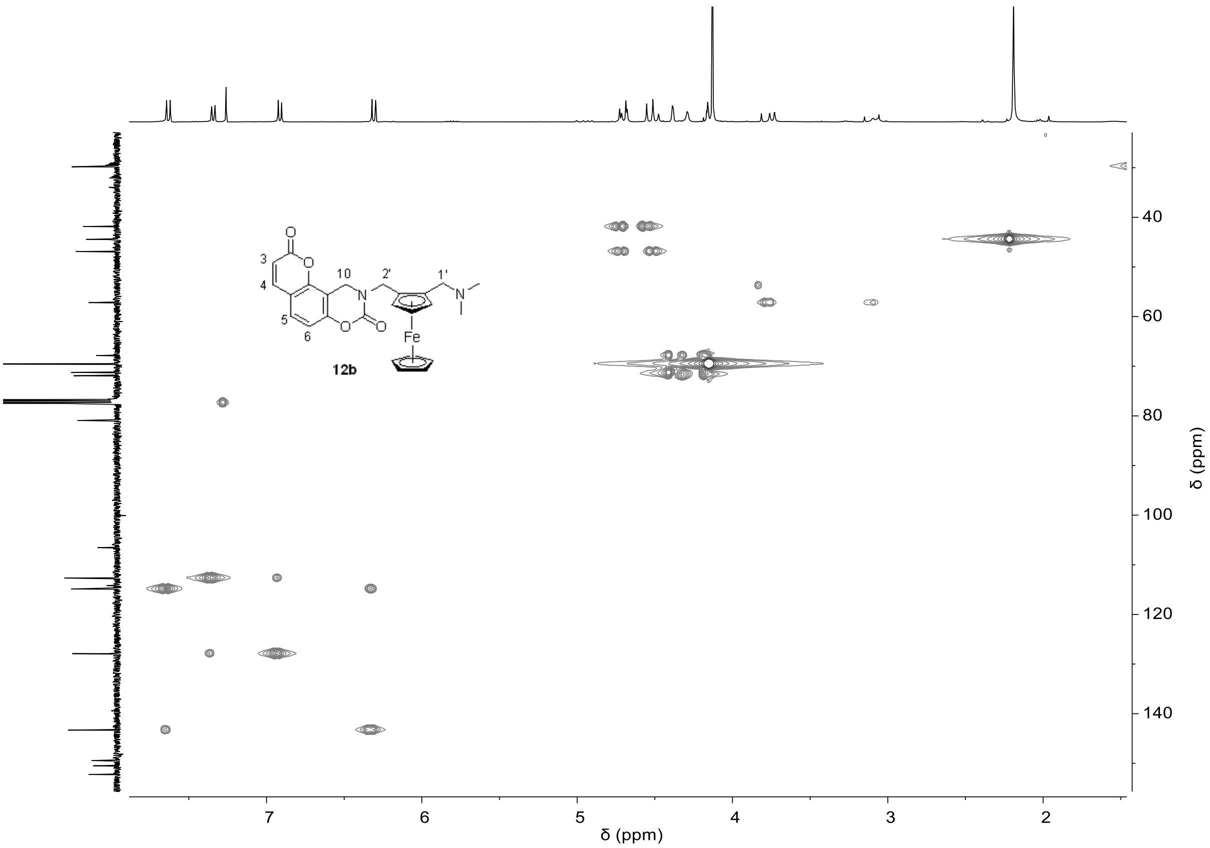

Representative HMBC spectrum of compound **12b**

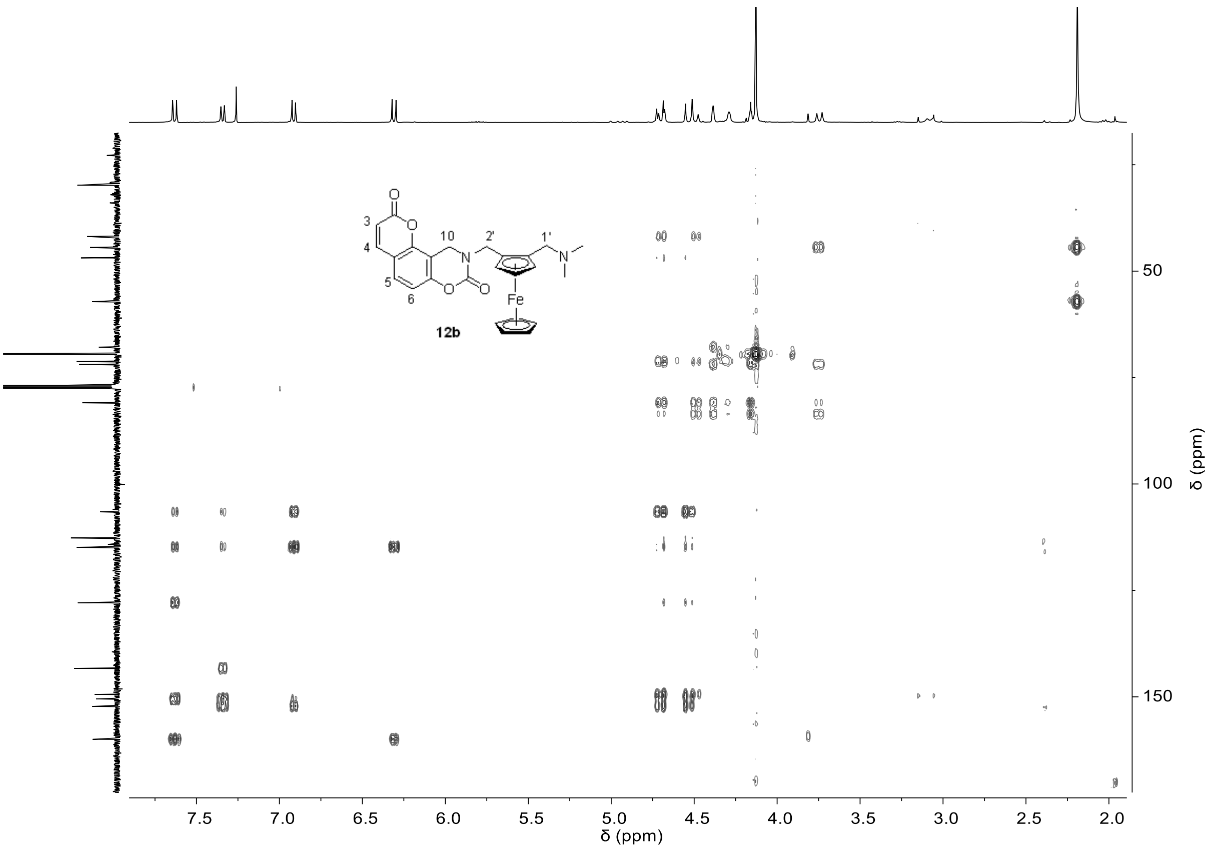

Representative COSY spectrum of compound **16d**

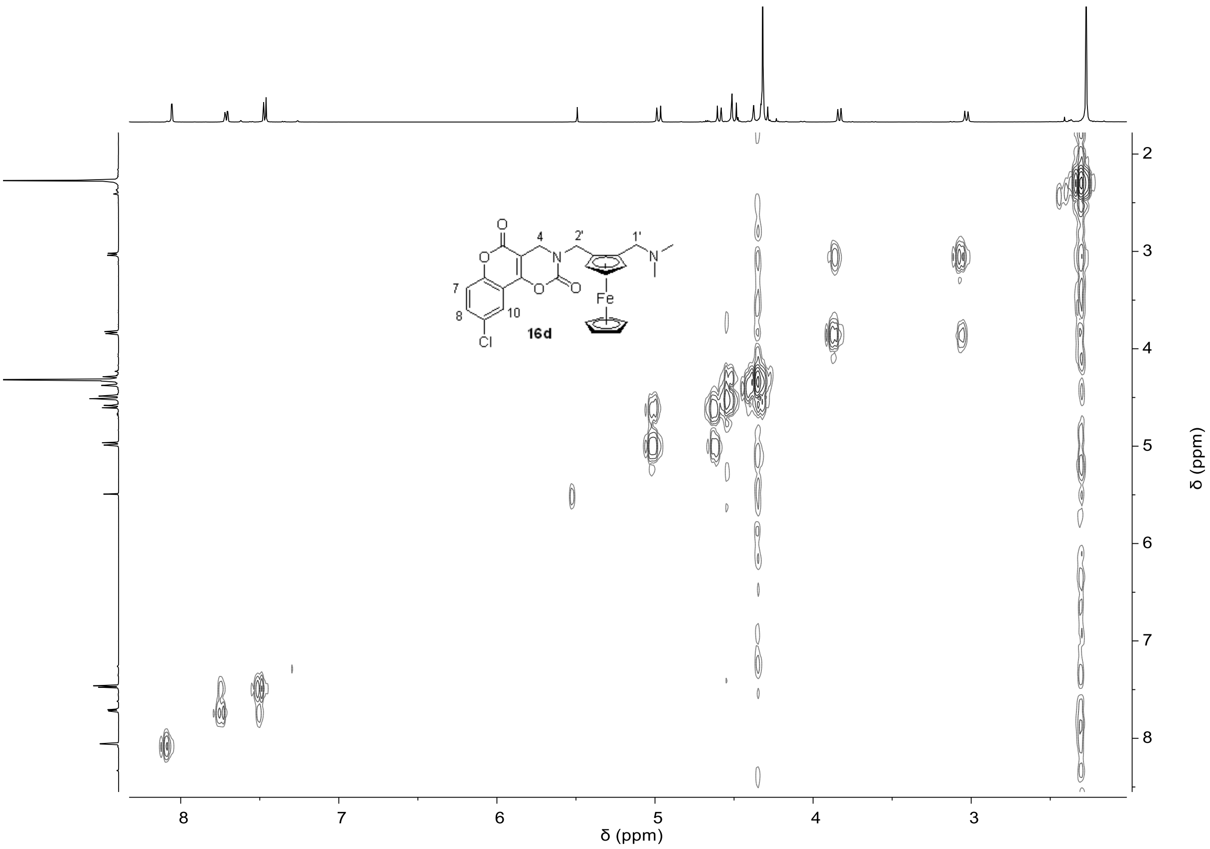

Representative HSQC spectrum of compound **16d**

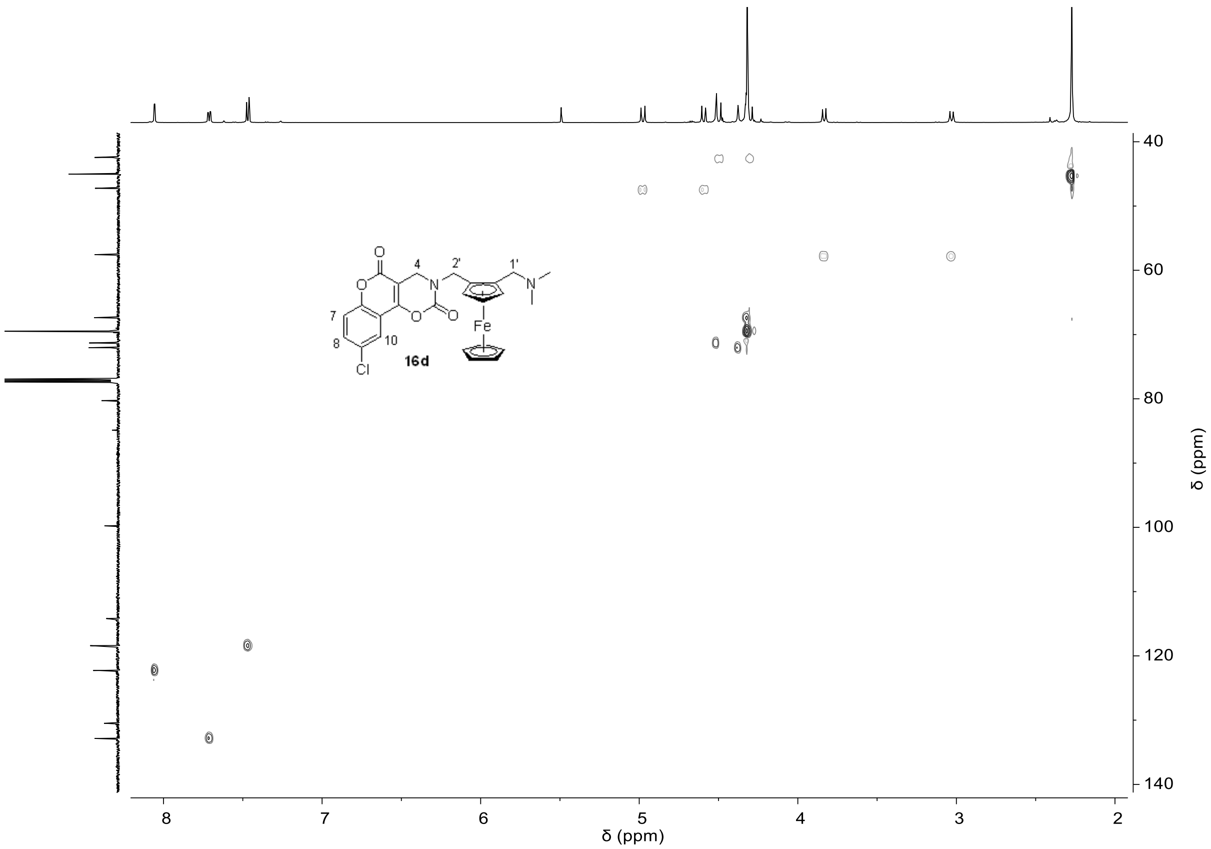

Representative HMBC spectrum of compound **16d**

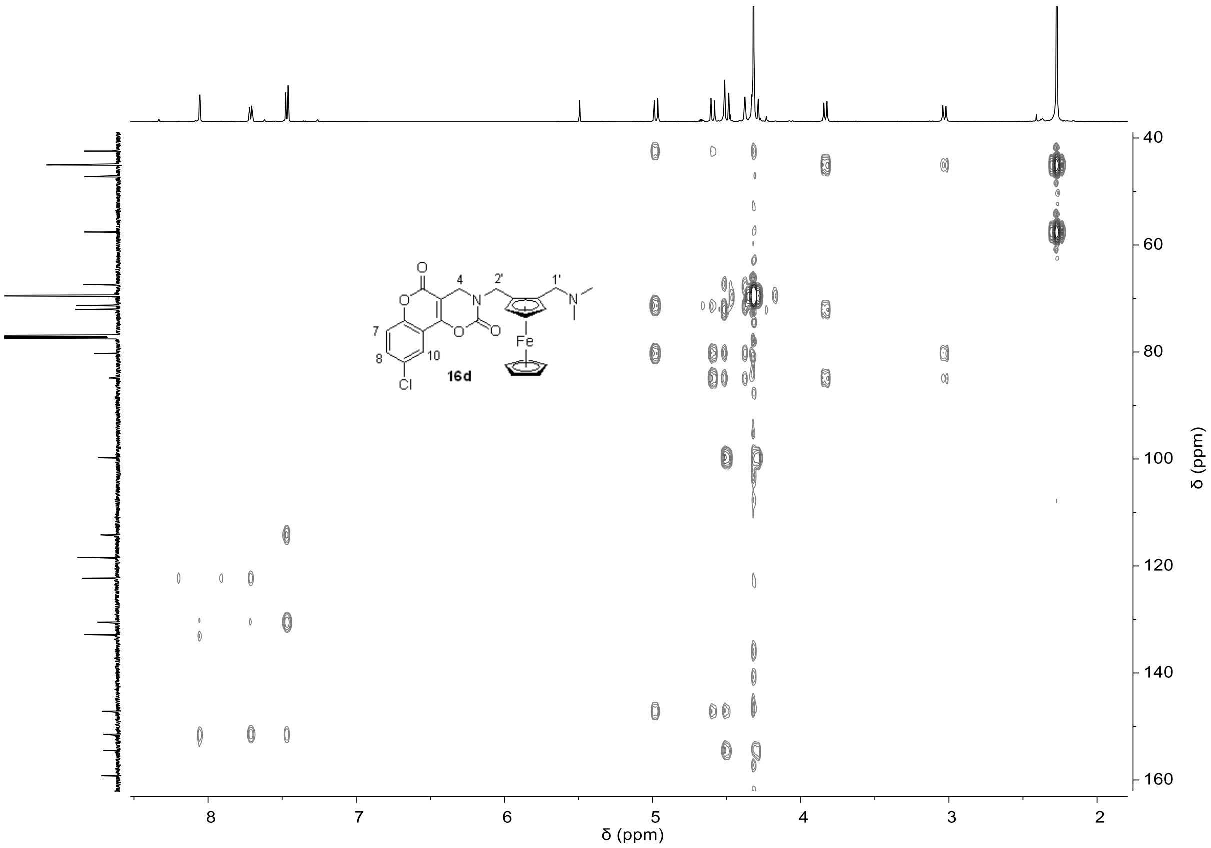

References

1. Beteck, R. M.; Legoabe, L. J.; Isaacs, M.; Khanye, S. D.; Laming, D.; Hoppe, H. C., Anti-trypanosomal and antimalarial properties of tetralone derivatives and structurally related benzocycloalkanones. *Medicina* **2019,** 55, (5), 206.
